# Aczel Alsina t-norm and t-conorm-based aggregation operators under linguistic interval-valued intuitionistic fuzzy setting with application

**DOI:** 10.7717/peerj-cs.1979

**Published:** 2024-05-27

**Authors:** Afra Siab, Muhammad Sajjad Ali Khan, Muhammad Asif Jan, Vladimir Simic, Nebojsa Bacanin, Tapan Senapati

**Affiliations:** 1 Institute of Numerical Sciences, Kohat University of Science and Technology, Kohat, Pakistan; 2 Department of Mathematics, Khushal Khan Khattak University, Karak, Pakistan; 3 Faculty of Transport and Traffic Engineering, University of Belgrade, Vojvode Stepe, Belgrade, Serbia; 4 Department of Industrial Engineering and Management, Yuan Ze University, Taoyuan City, Taiwan; 5 Faculty of Informatics and Computing, Singidunum University, Danijelova, Belgrade, Serbia; 6 School of Mathematics and Statistics, Southwest University, Beibei, Chongqing, China; 7 Saveetha School of Engineering, Saveetha Institute of Medical and Technical Sciences, Chennai, Tamilnadu, India

**Keywords:** Linguistic intuitionistic fuzzy numbers, Linguistic intuitionistic fuzzy Aczel-Alsina aggregation operators, Multi-criteria decision-making method

## Abstract

This article uses the Aczel-Alsina t-norm and t-conorm to make several new linguistic interval-valued intuitionistic fuzzy aggregation operators. First, we devised some rules for how linguistic interval-valued intuitionistic fuzzy numbers should work. Then, using these rules as a guide, we created a set of operators, such as linguistic interval-valued intuitionistic fuzzy Aczel-Alsina weighted averaging (LIVIFAAWA) operator, linguistic interval-valued intuitionistic fuzzy Aczel-Alsina weighted geometric (LIVIFAAWG) operator, linguistic interval-valued intuitionistic fuzzy Aczel-Alsina ordered weighted averaging (LIVIFAAOWA) operator, linguistic interval-valued intuitionistic fuzzy Aczel-Alsina ordered weighted geometric (LIVIFAAOWG) operator, linguistic interval-valued intuitionistic fuzzy Aczel-Alsina hybrid weighted averaging (LIVIFAAHWA) operator and linguistic interval-valued intuitionistic fuzzy Aczel-Alsina hybrid weighted geometric (LIVIFAAHWG) operators are created. Several desirable qualities of the newly created operators are thoroughly studied. Moreover, a multi-criteria group decision-making (MCGDM) method is proposed based on the developed operators. The proposed operators are then applied to real-world decision-making situations to demonstrate their applicability and validity to the reader. Finally, the suggested model is contrasted with the currently employed method of operation.

## Introduction

The concepts of uncertainty and ambiguity hold pivotal roles in the realms of science and mathematics, igniting recent fervour in mathematical circles. Originating in the late 19th century, uncertainty emerged as a significant concern, particularly evident in problems involving multi-criteria decision-making (MCDM). Throughout the 19th century, researchers encountered myriad obstacles in grappling with such challenges. Addressing the need for precise numerical values in resolving MCDM quandaries, Zadeh (1965) embarked on a pioneering investigation into the realm of uncertainty within practical fieldwork. Introducing the innovative notion of a fuzzy set, Zadeh sought to confront and mitigate the inherent uncertainty in MCDM scenarios. Unlike traditional sets with clear-cut boundaries, a fuzzy set embodies ambiguity, allowing for degrees of membership rather than binary acceptance or rejection. Due to fuzzy set theory, we now have the opportunity to communicate fuzzy concepts in meaningful ways using everyday language (Wang & Parkan, 2005; Zhang et al., 2024).

However, many realistic conditions pose limitations, including inadequate knowledge ambiguity, challenges in information collection, and more (Wu et al., 2018; Li et al., 2023; Chen et al., 2023). Determining characteristics with exact numbers presents challenges (Li et al., 2018). In later years, researchers built on the idea of a fuzzy set and named new types of fuzzy sets, such as type-1, type-2, the intuitionistic fuzzy number, the interval value intuitionistic fuzzy number, the hesitant fuzzy number (Zuo et al., 2023), and the Pythagorean hesitant fuzzy number (Zeng et al., 2024). Atanassov (1999) was the first to introduce the intuitionist fuzzy set (IFS). It is a very effective tool for dealing with information and is defined by a degree of membership or non-membership. Researchers from many fields have shown great interest in the IFS, and several developments have been made, including the IFS entropy measure (Burillo & Bustince, 1996), distance measure, and similarity measure (Hung & Yang, 2008).

Xu & Yager (2006) suggested using the ordered weighted aggregation operator to group the IF information by giving each input value a weight based on its position in the list. Additionally, several authors have devised extra ways to deal with unclear and wrong information when there are two or more sources of uncertainty simultaneously (Tan & Chen, 2010; Xu & Xia, 2011; Wan & Yi, 2015). Atanassov & Gargov (1989) eventually generalised it as an interval-valued intuitionist fuzzy set (IVIFS). IVIFS uses intervals instead of a single integer for its membership (MD) and non-membership (NMD) functions. There are several uses and implications of the IVIFS, notably in multi-criteria decision analysis (MCDA). Many research studies have used decision-making models and methods in the IVIFS environment. These have included operational laws (Luo, Xu & Gou, 2018), score functions (Dymova, Sevastjanov & Tikhonenko, 2013), accuracy values (Nayagam, Muralikrishnan & Sivaraman, 2011), preference linkages between sets (Chen & Xu, 2019), and different ways of choosing (Kumar & Garg, 2018) that depend on the sets. Even though IFSs and IVIFSs have grown in significance and acceptance within MCDA, they can only use numbers to represent the rating levels’ real numbers.

In contrast, practical decision-making problems may use other variables to denote the rating values, of which the most important types are linguistic variables (Shen et al., 2018; Gupta et al., 2018; Mao et al., 2019). Chen, Liu & Pei (2015) suggested linguistic IFS (LIFS) as a way to expand IFS. In LIFS, linguistic variables stand for MD and NMD. Other similar techniques supporting Because of such articulation, several researchers have applied the LIFSs to address decision-making problems (DMP). For instance, Li, Liu & Qin (2017) used LIFSs to define entropy and some new operational laws, and Zhang et al. (2017) used LIFSs to present a method of extended outranking for multi-criteria DMP(MCDMP); similarly, Garg & Kumar (2018), Peng, Wang & Cheng (2018), Liu, Liu & Merigó (2018), and Teng & Liu (2019) used LIFSs and developed some average operators.

Menger (1942) made the expression of LIFS even better and created linguistic interval valued intuitionistic fuzzy sets (LIVIFS), where linguistic variable intervals show the MD and NMD values. Because of the LIVIFS environment, experts can use intervals of linguistic phrases rather than just one to convey their preferences. *i.e*., with the LIVIFSs, experts have more freedom. With these types of features, Menger (1942) defined some based on operators using LIVIFNs and solved MAGDM problems using these operators, which involve a weighted average operator (WA), ordered WA operator (OWA), a hybrid average operator (HA), a weighted geometric operator (WG), an ordered WG operator (OWG), a hybrid geometric operator (HG). Garg (2016) was the first to present the idea of triangular norms (t-NMs). Which was later advanced by many researchers, for example, Einstein’s t-norm and t-conorm (Garg, 2017), Lukasiewicz’s t-norm and t-conorm (Venkatesan & Sriram, 2019), Archimedean t-norm and t-conorm (Garg & Kumar, 2019) *etc*. Wei et al. (2018) presented the characteristics and related features of t-norms.

With a significant emphasis on parameter activity and changeability, Aczél & Alsina (1982) introduced new techniques under the name Aczel-Alsina t-norm, t-conorm. A score-level fusion method, the Aczel-Alsina norm (AA t-NM), was created by Wei, Wang & Zhang (2011) to close the gap between imposters and actual persons while increasing their distance. The operational laws of Aczel-Alsina are significant mathematical techniques that come in handy when dealing with ambiguous or partial data. Senapati, Chen & Yager (2022) introduced “Aczel-Alsina aggregation operators and their application to intuitionistic fuzzy multiple attribute decision making”. Later, Aczel-Alsina aggregation operators were applied in several fuzzy contexts and applied it several real-life problems, like cyclone disaster assessment (Senapati et al., 2022), financial investment (Senapati, 2024, 2022), healthcare disposal method (Senapati et al., 2023b), “sustainable transportation sharing practices” (Senapati et al., 2023c), global partner selection (Senapati, Martínez & Chen, 2023), monetary system (Senapati et al., 2023a), disease diagnosis (Jabeen et al., 2023), electric car assessment (Hussain et al., 2023), investment strategy selection (Haq et al., 2022).

In this work, we propose several aggregation operators based on Aczel-Alsina techniques to address MADM problems in LIVIF settings. Our proposed technique and procedure can be summarized as follows:

- The proposed aggregation operators consist of linguistic interval-valued intuitionistic fuzzy Aczel-Alsina weighted averaging (LIVIFAAWA), linguistic interval-valued intuitionistic fuzzy Aczel-Alsina ordered weighted averaging (LIVIFAAOWA) and LIVIF Aczel-Alsina hybrid average (LIVIFAAHA), linguistic interval-valued intuitionistic fuzzy Aczel-Alsina weighted geometric (LIVIFAAWG), linguistic interval-valued intuitionistic fuzzy Aczel-Alsina ordered weighted geometric (LIVIFAAOWG) and LIVIF Aczel-Alsina hybrid geometric (LIVIFAAHG) operators.
- Examine the behaviour and characteristics of the operators.
- Create an algorithm to address problems with MADM in the LIVIF environment.
- Present a new MADM technique that incorporates the LIVIFAAWA and LIVIFAAWG operators.
- Demonstrate that the suggested method is both legitimate and better.

The structure of the rest of this article is as follows: “Preliminaries” delves into the basics of LIVIFS and Aczel-Alsina operators, providing insights into their characteristics, definitions, and operating principles. “Aczel-Alsina Operational Rules for Linguistic Interval-Valued Intuitionistic Fuzzy Numbers” introduces Aczel-Alsina operations in the context of LIVIFNs. “LIVIF Aczel-Alsina Average Aggregation Operators” examines several proposed operators, including the LIVIFAAWA operator, the LIVIFAAOWA operator, and the LIVIFAAHA operator. “LIVIF Aczel-Alsina Geometric Aggregation Operators” introduces the remaining consistent operators: LIVIFAAWG, LIVIFAAOWG, and LIVIFAAHG operators. In “MADM Approach Based With LIVIF Information”, we explore a MADM model using the LIVIFAAWA operator. “Numerical Example” employs an example to illustrate the suggested method. “Investigation on the Impact of Parameter $\Gamma$ on DM Outcomes” investigates the impact of parameters on decision-making outcomes. In “Comparison Analysis”, we compare the suggested approach with various other similar techniques that support the chosen method. Finally, “Conclusions” presents the conclusion.

## Preliminaries

In the following part, some basic ideas about LIVIFSs, operational laws for LIVIFNs and a review of the Aczel-Alsina t-norm and t-conorm are presented.

### Linguistic interval valued intuitionistic fuzzy set

**Definition 1** (Garg & Kumar, 2019) *If* $V = \{ {v_1},{v_2}, \ldots ,{v_k}\}$ *represent the universal set while* ${\hat S_{[0,q]}} = \{ {\hat s_\mu }|$ $0 \le \mu \le q\}$ *be a linguistic term set. The LIVIFS Z is defined as:*

$$Z = \{ \left(v,[\hat s_\theta ^ - \left(v\right),\hat s_\theta ^ + \left(v\right)],[\hat s_\beta ^ - \left(v\right),\hat s_\beta ^ + \left(v\right)]\right)|v \in V\} ,$$

*where the intervals* $[\hat s_\theta ^ - \left(v\right),\hat s_\theta ^ + \left(v\right)]$ *and* $[\hat s_\beta ^ - \left(v\right),\hat s_\beta ^ + \left(v\right)]$ *demonstrate the MD and NMD values, respectively, and for each* $v \in V,$ *the pair* $\left([\hat s_\theta ^ - \left(v\right),\hat s_\theta ^ + \left(v\right)],[\hat s_\beta ^ - \left(v\right),\hat s_\beta ^ + \left(v\right)]\right)$ *with the condition* $\hat s_\theta ^ + \left(v\right) + \hat s_\beta ^ + \left(v\right) \le q$ *is known as LIVIFN*.

**Definition 2** *For any* $v \in V$ *the extent of the linguistic indeterminacy of* $v$ *to Z is denoted by* ${\hat s_{\pi \left(v\right)}}$ *and defined as* $:{\hat s_{\pi \left(v\right)}} = [\hat s_{\pi \left(v\right)}^ - ,\hat s_{\pi \left(v\right)}^ + ] = [q - \hat s_\theta ^ + \left(v\right) - \hat s_\beta ^ + \left(v\right),q - \hat s_\theta ^ - \left(v\right) - \hat s_\beta ^ - \left(v\right)].$

**Definition 3** (Schweiser, 1961) *Let* ${Z_1} = \left([\hat s_{{\theta _1}}^ - ,\hat s_{{\theta _1}}^ + ],[\hat s_{{\beta _1}}^ - ,\hat s_{{\beta _1}}^ + ]\right)$, ${Z_2} = \left([\hat s_{{\theta _2}}^ - ,\hat s_{{\theta _2}}^ + ],[\hat s_{{\beta _2}}^ - ,\hat s_{{\beta _2}}^ + ]\right)$ *be LIVIFNs, such that* $\hat s_{{\theta _1}}^ - ,\hat s_{{\theta _1}}^ + ,$ $\hat s_{{\beta _1}}^ - ,\hat s_{{\beta _1}}^ + ,\hat s_{{\theta _2}}^ - ,\hat s_{{\theta _2}}^ + ,\hat s_{{\beta _2}}^ - ,\hat s_{{\beta _2}}^ + \in {\hat S_{[0,q]}}$ *and* $\Gamma \,\gt\, 0$, *the operational laws are held as defined below:*

$$\eqalign{{Z_1} \oplus {Z_2} = &  \left( {\matrix{ {\left[ {{{\hat s}_{q\left( {{{\theta _1^ - } \over q} + {{\theta _2^ - } \over q} - {{\theta _1^ - \theta _2^ - } \over {{q^2}}}} \right)}},{{\hat s}_{q\left( {{{\theta _1^ + } \over q} + {{\theta _2^ + } \over q} - {{\theta _1^ + \theta _2^ + } \over {{q^2}}}} \right)}}} \right],\left[ {{{\hat s}_{q\left( {{{\beta _1^ - \beta _2^ - } \over {{q^2}}}} \right)}},{{\hat s}_{q\left( {{{\beta _1^ + \beta _2^ + } \over {{q^2}}}} \right)}}} \right]}} } \right),\cr {Z_1} \otimes {Z_2} = &  \left( {\matrix{{\left[ {{{\hat s}_{q\left({{\theta _1^ - \theta _2^ - } \over {{q^2}}}\right)}},{{\hat s}_{q\left({{\theta _1^ + \theta _2^ + } \over {{q^2}}}\right)}}} \right],\left[ {{{\hat s}_{q\left( {{{\beta _1^ - } \over q} + {{\beta _2^ - } \over q} - {{\beta _1^ - \beta _2^ - } \over {{q^2}}}} \right)}},{{\hat s}_{q\left( {{{\beta _1^ + } \over q} + {{\beta _2^ + } \over q} - {{\beta _1^ + \beta _2^ + } \over {{q^2}}}} \right)}}} \right]}} } \right),\cr \Gamma {Z_1} = &  \left( {\left[ {{{\hat s}_{q\left( {1 - {{\left(1 - {{\theta _1^ - } \over q}\right)}\Gamma }} \right)}},{{\hat s}_{q\left( {1 - {{\left(1 - {{\theta _1^ + } \over q}\right)}\Gamma }} \right)}}} \right],\left[ {{{\hat s}_{q{{\left({{\beta _1^ - } \over q}\right)}\Gamma }}},{{\hat s}_{q{{\left({{\beta _1^ + } \over q}\right)}\Gamma }}}} \right]} \right),\cr Z_1^\Gamma = &  \left( {\left[ {{{\hat s}_{q{{\left( {{{\theta _1^ - } \over q}} \right)}\Gamma }}},{{\hat s}_{q{{\left( {{{\theta _1^ + } \over q}} \right)}\Gamma }}}} \right],\left[ {{{\hat s}_{q\left( {1 - {{\left(1 - {{\beta _1^ - } \over q}\right)}\Gamma }} \right)}},{{\hat s}_{q\left( {1 - {{\left(1 - {{\beta _1^ + } \over q}\right)}\Gamma }} \right)}}} \right]} \right).}$$

For the comparison of LIVIFNs, Garg & Kumar (2019) defined the score and accuracy functions, as below.

**Definition 4** (Garg & Kumar, 2019) *Let* $Z = \left([\hat s_\theta ^ - ,\hat s_\theta ^ + ],[\hat s_\beta ^ - ,\hat s_\beta ^ + ]\right)$ *be a LIVIFN, then the score function of Z denoted* ${\widetilde{S}}\left( Z \right)$ *and defined as* ${\widetilde{S}}\left( Z \right) = {\widetilde{s}}_{{{\left(2q + {\theta ^ - } + {\theta ^ + } - {\beta ^ - } - {\beta ^ + }\right)} \over 4}.}$

*Likewise, the accuracy function of Z is represented by* ${\widetilde{H}}\left(Z\right)$ *and defined as follows:*

$${\widetilde{H}}\left(Z\right) = {\widetilde{s}}_{{{\left( {{\theta ^ - } + {\theta ^ + } + {\beta ^ - } + {\beta ^ + }} \right)} \over 2}}.$$

Any two LIVIFNs, ${Z_1} = \left([\hat s_{{\theta _1}}^ - ,\hat s_{{\theta _1}}^ + ],[\hat s_{{\beta _1}}^ - ,\hat s_{{\beta _1}}^ + ]\right)$ and ${Z_2} = \left([\hat s_{{\theta _2}}^ - ,\hat s_{{\theta _2}}^ + ],[\hat s_{{\beta _2}}^ - ,\hat s_{{\beta _2}}^ + ]\right)$, can be ranked by the following way:

**(i)** If ${\widetilde{S}}\left({Z_1}\right)\;\lt\;{\widetilde{S}}\left({Z_2}\right)$, this implies that ${Z_1}\;\lt\;{Z_2}$;
**(ii)** If ${\widetilde{S}}\left( {{Z_1}} \right) = {\widetilde{S}}\left( {{Z_2}} \right)$, this implies that ${Z_1} = {Z_2}$;
**(iii)** If ${\widetilde{H}}\left( {{Z_1}} \right) = {\widetilde{H}}\left( {{Z_2}} \right)$, this implies that ${Z_1} = {Z_2}$;
**(iv)** If ${\widetilde{H}}\left( {{Z_1}} \right)\;\lt\; {\widetilde{H}}\left( {{Z_2}} \right)$, this implies that ${Z_1}\;\lt\;{Z_2}$;
**(v)** If ${\widetilde{H}}\left({Z_1}\right) \;\gt\; {\widetilde{H}}\left({Z_2}\right)$, this implies that ${Z_1} \;\gt\; {Z_2}$.

In Teng & Liu (2019), the writers created the concept of entropy for IVLIFN. We adopt the same idea for Linguistic interval-valued intuitionistic fuzzy numbers:

**Definition 5** (Wei et al., 2018) *An entropy function* $N:LIVIFS\left(V\right) \to [0,1]$ *which satisfies the following axioms:*

1. *N* $\left(A\right)$ $= 0$ *if and only if A is a crisp set;*
2. *N* $\left(A\right)$ $= 1$ *if and only if* $[\hat s_{{\theta _A}}^ - \left({v_i}\right),\hat s_{{\theta _A}}^ + \left({v_i}\right)] = [\hat s_{{\beta _A}}^ - \left({v_i}\right),\hat s_{{\beta _A}}^ + \left({v_i}\right)]$ *for each* ${v_i} \in V$ ;
3. *N* $\left(A\right)$ $\le$ *N* $\left(B\right)$, *if A is less fuzzy than B;*
4. $N\left(A\right) = N\left({A^c}\right).$

*and is defined as;*

(1) $$N = {1 \over n}\sum\limits_{i = 1}^n {{{2{q^2} - {{\left| {\hat s_{{\theta _A}}^ + \left({v_i}\right) - \hat s_{{\beta _A}}^ + \left({v_i}\right)} \right|}^2} - {{\left| {\hat s_{{\theta _A}}^ - \left({v_i}\right) - \hat s_{{\beta _A}}^ - \left({v_i}\right)} \right|}^2} + {{\left(\hat s_{{\pi _A}}^ - \left({v_i}\right)\right)}^2} + {{\left(\hat s_{{\pi _A}}^ + \left({v_i}\right)\right)}^2}} \over {4{q^2}}}}$$

### T-NM, T-CNM, Aczel-Alsina T-NM

The concept of t-NM was initially presented by Schweizer & Sklar (1960) as an extension of Peng, Wang & Cheng (2018) theories. In the case of probabilistic metric spaces, they established their approach to generalise the triangle inequality of metrics; however, their approach has been tested in various fields, including fuzzy set theory. Originally investigated in the setting of probabilistic metric spaces, the t-conorms (dual operations to t-NMs) was subsequently expanded to include fuzzy disjunctions (Schweiser, 1961).

**Definition 6** (Aczél & Alsina, 1982; Schweizer & Sklar, 1960) *If* $T:{[0,1]^2} \to [0,1]$ *is a function. Its neutral element is* $e = 1$ *i.e.*, $T\left(v,1\right) = T\left(1,v\right) = v$ *for all* $v \in [0,1]$, *T is called a t-NM. Likewise, if its neutral element is* $e = 0$ *i.e.*, $T\left(v,0\right) = T\left(0,v\right) = v$ *for all* $v \in [0,1]$, *then T is called a t-CNM*.

To distinguish t-NMs from t-CNMs with clarity, *T* will be used as usual t-norms, and *S* will be used t-conorms. A t-norms is represented by the function $S:{[0,1]^2} \to [0,1]$ defined as $S\left(v,u\right) = 1 - T\left(1 - v,1 - u\right)$ while a t-norms is $T:{[0,1]^2} \to [0,1]$ defined as $T\left(v,u\right) = 1 - S\left(1 - v,1 - u\right)$.

It is easy to see that ${T_M}\left(v,u\right) = min\left(v,u\right)$ is the strongest (largest) t-NM using the notation from Garg (2016), while drastic product ${T_D}$ is the smallest t-NM, which vanishes on $[0,1)^2$ (obviously, if $max\left(v,u\right) = 1$, then $T\left(v,u\right)$ $= min\left(v,u\right)$ for any t-NM). The Lukasiewicz t-NM denoted by ${T_L},$ defined as ${T_L}\left(v,u\right) = max\left(0,v + u - 1\right)$ and two traditional t-NMs that make up the product t-NM *TP* play a significant role in both theory and applications. See Aczél & Alsina (1982) for more information and thorough findings. We’ll look at some of the most unique t-NMs in this article. Like rigorous t-NMs, these are produced by reducing the bijective additive generators and invariant to the t-NM $t:[0,1] \to [0,\infty ]$. $T\left(v,u\right) = {t^{ - 1}}\left(t\left(v\right) + t\left(u\right)\right)$ Taking into account the n-array expansion in this instance, $T\left({v_1}, \ldots ,{v_m}\right) = {t^{ - 1}}\left(\sum\nolimits_{j = 1}^m t \left({v_i}\right)\right).$ The product t-NM ${T_P},$ extremal t-NMs ${T_M}$ and ${T_D}$, commute with the power functions, satisfying the equivalence $T\left({v^\alpha },{u^\alpha }\right) = T{\left(v,u\right)^\alpha }$ for all $\alpha \,\gt\, 0$. Aczél & Alsina (1982) reported tnorm solutions aforementioned in the early 1980s (Garg, 2017), implying that ${t_\psi },\psi \in ]0,\infty [$ provided by ${t_\psi }\left(v\right) = {\left( - \log v\right)^\psi }$. $T_A^\psi$ are defined as:

$$T_A^\psi \left(v,u\right) = \left\{ \matrix{ {{T_D}\left(v,u\right),}\hfill & {{\mathrm{ if }}\;\psi = 0}\hfill \cr {\min \left(v,u\right),}\hfill &  {{\mathrm{ if }}\;\psi = \infty } \hfill \cr {{e^{ - {{\left({{\left( - \log v\right)}^\psi } + {{\left( - \log u\right)}^\psi }\right)}^{{1 \over \psi }}}}},}\hfill &  {{\mathrm{ otherwise.}}}\hfill}\right.$$

The extremal t-NMs, which are strictly increasing and continuous in parameter, are added to create the Aczel-Alsina family $\left(T_A^\psi \right),$ $\psi \in [0,\infty ]$ of t-NMs.

Due to their dual nature, t-CNMs may be used to make similar statements and provide examples. ${S_M} = \max$(dual to ${T_M}$) is lowest tconorm and drastic ${S_D}$ is the greatest, which is always 1 on $(0,1]^2$. If $\min \left(v,u\right) = 0$ and *S* is any t-CNM, then $S\left(v,u\right) = \max \left(v,u\right)$. The ${T_L}$ has a dual t-CNM denoted by ${S_L}$ and defined as ${S_L}\left(v,u\right) = \min \left(1,v + u\right)$, and ${T_P}$ has a dual t-CNM denoted by ${S_P}$ is given by ${S_P}\left(v,u\right) = v + u - vu$. Additionally, additive generators (which are growing) may be used to produce continuous Archimedean t-CNMs. If *S* is dual to a continuous Archimedean t-NM *T* produced by an additive generator, then *S* is produced by an additive generator $t$ supplied by $S\left(v\right) = t\left(1 - v\right)$. $S_A^\psi$ are defined as:

$$S_A^\psi \left(v,u\right) = \left\{\matrix{ {{S_D}\left(v,u\right),}\hfill &  {{\mathrm{ if }}\;\psi = 0}\hfill \cr {\min \left(v,u\right),}\hfill &  {{\mathrm{ if }}\;\psi = \infty }\hfill \cr {1 - {e^{ - {{\left({{\left( - \log \left(1 - v\right)\right)}^\psi } + {{\left( - \log \left(1 - u\right)\right)}^\psi }\right)}^{{1 \over \psi }}}}},}\hfill &  {{\mathrm{ otherwise}}}\hfill}\right.$$

When the extremal t-CNMs are added, the Aczel-Alsina family $\left(S_A^\psi \right),\psi \in \left[ {0,\infty } \right]$ of t-CNMs is obtained.

## Aczel-alsina operational rules for linguistic interval-valued intuitionistic fuzzy numbers

Next, we will apply the Aczel-Alsina t-norm and t-conorm to define Aczel-Alsina operational laws for LIVIFNs.

**Definition 7** *Let* ${Z_1} = \left([\hat s_{{\theta _1}}^ - ,\hat s_{{\theta _1}}^ + ],[\hat s_{{\beta _1}}^ - ,\hat s_{{\beta _1}}^ + ]\right)$, ${Z_2} = \left([\hat s_{{\theta _2}}^ - ,\hat s_{{\theta _2}}^ + ],[\hat s_{{\beta _2}}^ - ,\hat s_{{\beta _2}}^ + ]\right)$ *be two LIVIFNs, and* $\Gamma$, $\tau\, > \,0$. *Then, the operational laws for LIVIFNs are defined as following:*

$$\left( a \right)\,{Z_1} \oplus {Z_2} = \left( {\matrix{ {\left[ {{{\hat s}_{q\left( {1 - {e^{ - {{\left\langle {{{\left( - \log \left(1 - {{\theta _1^ - } \over q}\right)\right)}\Gamma } + {{\left( - \log \left(1 - {{\theta _2^ - } \over q}\right)\right)}\Gamma }} \right\rangle }^{{1 \over \Gamma }}}}}} \right)}},{{\hat s}_{q\left( {1 - {e^{ - {{\left\langle {{{\left( - \log \left(1 - {{\theta _1^ + } \over q}\right)\right)}\Gamma } + {{\left( - \log \left(1 - {{\theta _2^ + } \over q}\right)\right)}\Gamma }} \right\rangle }^{{1 \over \Gamma }}}}}} \right)}}} \right],} \cr  {\left[ {{{\hat s}_{q\left( {{e^{ - {{\left\langle {{{\left( - \log \left({{\beta _1^ - } \over q}\right)\right)}\Gamma } + {{\left( - \log \left({{\beta _2^ - } \over q}\right)\right)}\Gamma }} \right\rangle }^{{1 \over \Gamma }}}}}} \right)}},{{\hat s}_{q\left( {{e^{ - {{\left\langle {{{\left( - \log \left({{\beta _1^ + } \over q}\right)\right)}\Gamma } + {{\left( - \log \left({{\beta _2^ + } \over q}\right)\right)}\Gamma }} \right\rangle }^{{1 \over \Gamma }}}}}} \right)}}} \right]} \cr  } } \right);$$

$$\left( b \right)\,{Z_1} \otimes {Z_2} = \left( {\matrix{ {\left[ {{{\hat s}_{q\left( {{e^{ - {{\left\langle {{{\left( - \log \left({{\theta _1^ - } \over q}\right)\right)}\Gamma } + {{\left( - \log \left({{\theta _2^ - } \over q}\right)\right)}\Gamma }} \right\rangle }^{{1 \over \Gamma }}}}}} \right)}},{{\hat s}_{q\left( {{e^{ - {{\left\langle {{{\left( - \log \left({{\theta _1^ + } \over q}\right)\right)}\Gamma } + {{\left( - \log \left({{\theta _2^ + } \over q}\right)\right)}\Gamma }} \right\rangle }^{{1 \over \Gamma }}}}}} \right)}}} \right],} \cr  {\left[ {{{\hat s}_{q\left( {1 - {e^{ - {{\left\langle {{{\left( - \log \left(1 - {{\beta _1^ - } \over q}\right)\right)}\Gamma } + {{\left( - \log \left(1 - {{\beta _2^ - } \over q}\right)\right)}\Gamma }} \right\rangle }^{{1 \over \Gamma }}}}}} \right)}},{{\hat s}_{q\left( {1 - {e^{ - {{\left\langle {{{\left( - \log \left(1 - {{\beta _1^ + } \over q}\right)\right)}\Gamma } + {{\left( - \log \left(1 - {{\beta _2^ + } \over q}\right)\right)}\Gamma }} \right\rangle }^{{1 \over \Gamma }}}}}} \right)}}} \right]} \cr  } } \right);$$

$$\left( c \right)\,\tau {Z_1} = \left( {\matrix{ {\left[ {{{\hat s}_{q\left( {1 - {e^{ - {{\left\langle {\tau {{\left( - \log \left(1 - {{\theta _1^ - } \over q}\right)\right)}\Gamma }} \right\rangle }^{{1 \over \Gamma }}}}}} \right)}},{{\hat s}_{q\left( {1 - {e^{ - {{\left\langle {\tau {{\left( - \log \left(1 - {{\theta _1^ + } \over q}\right)\right)}\Gamma }} \right\rangle }^{{1 \over \Gamma }}}}}} \right)}}} \right],} \cr  {\left[ {{{\hat s}_{q\left( {{e^{ - {{\left\langle {\tau {{\left( - \log \left({{\beta _1^ - } \over q}\right)\right)}\Gamma }} \right\rangle }^{{1 \over \Gamma }}}}}} \right)}},{{\hat s}_{q\left( {{e^{ - {{\left\langle {\tau {{\left( - \log \left({{\beta _1^ + } \over q}\right)\right)}\Gamma }} \right\rangle }^{{1 \over \Gamma }}}}}} \right)}}} \right]} \cr  } } \right);$$

$$\left( d \right)\,Z_1^\tau = \left( {\matrix{ {\left[ {{{\hat s}_{q\left( {{e^{ - {{\left\langle {\tau {{\left( - \log \left({{\theta _1^ - } \over q}\right)\right)}\Gamma }} \right\rangle }^{{1 \over \Gamma }}}}}} \right)}},{{\hat s}_{q\left( {{e^{ - {{\left\langle {\tau {{\left( - \log \left({{\theta _1^ + } \over q}\right)\right)}\Gamma }} \right\rangle }^{{1 \over \Gamma }}}}}} \right)}}} \right],} \cr  {\left[ {{{\hat s}_{q\left( {1 - {e^{ - {{\left\langle {\tau {{\left( - \log \left(1 - {{\beta _1^ - } \over q}\right)\right)}\Gamma }} \right\rangle }^{{1 \over \Gamma }}}}}} \right)}},{{\hat s}_{q\left( {1 - {e^{ - {{\left\langle {\tau {{\left( - \log \left(1 - {{\beta _1^ + } \over q}\right)\right)}\Gamma }} \right\rangle }^{{1 \over \Gamma }}}}}} \right)}}} \right]} \cr  } } \right).$$

**Example 1** *Let* ${Z_1} = \left([{\hat s_1},{\hat s_3}],[{\hat s_2},{\hat s_3}]\right)$ *and* ${Z_2} = \left([{\hat s_2},{\hat s_4}],[{\hat s_1},{\hat s_2}]\right)$ *be two LIVIFNs, and* $\Gamma = 2,\,\tau = 3$ *and* $l = 6.$

$$\eqalign{\left( a \right)\,{Z_1} \oplus {Z_2} = &  \left( {\left[ {{{\hat s}_{6\left( {1 - {e^{ - {{\left\langle {{{\left( - \log \left(1 - {1 \over 6}\right)\right)}^2} + {{\left( - \log \left(1 - {2 \over 6}\right)\right)}^2}} \right\rangle }^{{1 \over 2}}}}}} \right)}},{{\hat s}_{6\left( {1 - {e^{ - {{\left\langle {{{\left( - \log \left(1 - {3 \over 6}\right)\right)}^2} + {{\left( - \log \left(1 - {4 \over 6}\right)\right)}^2}} \right\rangle }^{{1 \over 2}}}}}} \right)}}} \right]} \right.,\\  & \left. {\left[ {{{\hat s}_{6\left( {{e^{ - {{\left\langle {{{\left( - \log \left({2 \over 6}\right)\right)}^2} + {{\left( - \log \left({1 \over 6}\right)\right)}^2}} \right\rangle }^{{1 \over 2}}}}}} \right)}},{{\hat s}_{6\left( {{e^{ - {{\left\langle {{{\left( - \log \left({3 \over 6}\right)\right)}^2} + \big( - \log {{\left({2 \over 6}\right)}^2}}. \right\rangle }^{{1 \over 2}}}}}} \right)}}} \right]} \right)\\ = &  (\left[ {{{\hat s}_{2.1534}},{{\hat s}_{4.3632}}} \right],\left[ {{{\hat s}_{0.7334}},{{\hat s}_{1.6368}}} \right]),}$$

$$\eqalign{\left( b \right)\,{Z_1} \otimes {Z_2}=&  \left( {\left[ {{{\hat s}_{6\left( {{e^{ - {{\left\langle {{{\left( - \log \left({1 \over 6}\right)\right)}^2} + {{\left( - \log \left({2 \over 6}\right)\right)}^2}} \right\rangle }^{{1 \over 2}}}}}} \right)}},{{\hat s}_{6\left( {{e^{ - {{\left\langle {{{\left( - \log \left({3 \over 6}\right)\right)}^2} + {{\left( - \log \left({4 \over 6}\right)\right)}^2}} \right\rangle }^{{1 \over 2}}}}}} \right)}}} \right]} \right.,\\ & \left. {\left[ { {\widetilde {s}}{_{6\left( {1 - {e^{ - {{\left\langle {{{\left( - \log \left(1 - {2 \over 6}\right)\right)}^2} + {{\left( - \log \left(1 - {1 \over 6}\right)\right)}^2}} \right\rangle }^{{1 \over 2}}}}}} \right)}},{ {\widetilde {s}}}{_{6\left( {1 - {e^{ - {{\left\langle {{{\left( - \log \left(1 - {3 \over 6}\right)\right)}^2} + {{\left( - \log \left(1 - {2 \over 6}\right)\right)}^2}} \right\rangle }^{{1 \over 2}}}}}} \right)}}} \right]} \right)\\= &  \left(\left[ {{{\hat s}_{0.7334}},{{\hat s}_{2.6878}}} \right],\left[ {{{\hat s}_{2.1534}},{{\hat s}_{3.3121}}} \right]\right),}$$

$$\eqalign{\left( c \right)\,3{Z_1}=  & \left( {\left[ {{{\hat s}_{6\left( {1 - {e^{ - {{\left\langle {3{{\left( - \log \left(1 - {1 \over 6}\right)\right)}^2}} \right\rangle }^{{1 \over 2}}}}}} \right)}},{{\hat s}_{6\left( {1 - {e^{ - {{\left\langle {3{{\left( - \log \left(1 - {3 \over 6}\right)\right)}^2}} \right\rangle }^{{1 \over 2}}}}}} \right)}}} \right]} \right.,\\ &\left. {\left[ {{{\hat s}_{6\left( {{e^{ - {{\left\langle {3{{\left( - \log \left({2 \over 6}\right)\right)}^2}} \right\rangle }^{{1 \over 2}}}}}} \right)}},{{\hat s}_{6\left( {{e^{ - {{\left\langle {3{{\left( - \log \left({3 \over 6}\right)\right)}^2}} \right\rangle }^{{1 \over 2}}}}}} \right)}}} \right]} \right)\\ =& \left(\left[ {{{\hat s}_{1.6247}},{{\hat s}_{4.1938}}} \right],\left[ {{{\hat s}_{0.8948}},{{\hat s}_{1.8061}}} \right]\right),}$$

$$\eqalign{\left( d \right)\, Z_1^3= &  \left( {\left[ {{{\hat s}_{6\left( {{e^{ - {{\left\langle {3{{\left( - \log \left({1 \over 6}\right)\right)}^2}} \right\rangle }^{{1 \over 2}}}}}} \right)}},{{\hat s}_{6\left( {{e^{ - {{\left\langle {3{{\left( - \log \left({3 \over 6}\right)\right)}^2}} \right\rangle }^{{1 \over 2}}}}}} \right)}}} \right]} \right.,\\  & \left. {\left[ {{{\hat s}_{6\left( {1 - {e^{ - {{\left\langle {3{{\left( - \log \left(1 - {2 \over 6}\right)\right)}^2}} \right\rangle }^{{1 \over 2}}}}}} \right)}},{{\hat s}_{6\left( {1 - {e^{ - {{\left\langle {3{{\left( - \log \left(1 - {3 \over 6}\right)\right)}^2}} \right\rangle }^{{1 \over 2}}}}}} \right)}}} \right]} \right)\\ = &  \left(\left[ {{{\hat s}_{0.2694}},{{\hat s}_{1.8061}}} \right],\left[ {{{\hat s}_{3.0273}},{{\hat s}_{4.1938}}} \right]\right).}$$

**Theorem 1** *Let* ${Z_1} = \left([\hat s_{{\theta _1}}^ - ,\hat s_{{\theta _1}}^ + ],[\hat s_{{\beta _1}}^ - ,\hat s_{{\beta _1}}^ + ]\right)$ *and* ${Z_2} = \left([\hat s_{{\theta _2}}^ - ,\hat s_{{\theta _2}}^ + ],[\hat s_{{\beta _2}}^ - ,\hat s_{{\beta _2}}^ + ]\right)$ *be two LIVIFNs, and* $\alpha ,{\alpha _1},{\alpha _2}\, > \,0$, *then*

**(i)** ${Z_1} \oplus {Z_2} = {Z_2} \oplus {Z_1}$;
**(ii)** ${Z_1} \otimes {Z_2} = {Z_2} \otimes {Z_1}$;
**(iii)** $\alpha \left({Z_1} \oplus {Z_2}\right) = \alpha {Z_2} \oplus \alpha {Z_1}$;
**(iv)** $\left({\alpha _1} + {\alpha _2}\right){Z_1} = {\alpha _1}{Z_1} \oplus {\alpha _2}{Z_2}$;
**(v)** ${\left({Z_1} \otimes {Z_2}\right)^\alpha } = Z_1^\alpha \otimes Z_2^\alpha$;
**(vi)** $Z_1^{{\alpha _1}} \otimes Z_1^{{\alpha _2}} = Z_1^{\left({\alpha _1} + {\alpha _2}\right)}$.

**Proof:** According to Definition 7, we have that

$$\eqalign{\left(i\right)\,{Z_1} \oplus {Z_2}=&  \left( \left[{{{{{\hat s}_{q\left( {1 - {e^{ - {{\left\langle {{{\left( - \log \left(1 - {{\theta _1^ - } \over q}\right)\right)}\Gamma } + {{\left( - \log \left(1 - {{\theta _2^ - } \over q}\right)\right)}\Gamma }} \right\rangle }^{{1 \over \Gamma }}}}}} \right)}},{{\hat s}_{q\left( {1 - {e^{ - {{\left\langle {{{\left( - \log \left(1 - {{\theta _1^ + } \over q}\right)\right)}\Gamma } + {{\left( - \log \left(1 - {{\theta _2^ + } \over q}\right)\right)}\Gamma }} \right\rangle }^{{1 \over \Gamma }}}}}} \right)}}} }}\right] \right.,\cr & \left. {\left[ {{{\hat s}_{q\left( {{e^{ - {{\left\langle {{{\left( - \log \left({{\beta _1^ - } \over q}\right)\right)}\Gamma } + {{\left( - \log \left({{\beta _2^ - } \over q}\right)\right)}\Gamma }} \right\rangle }^{{1 \over \Gamma }}}}}} \right)}},{{\hat s}_{q\left( {{e^{ - {{\left\langle {{{\left( - \log \left({{\beta _1^ + } \over q}\right)\right)}\Gamma } + {{\left( - \log \left({{\beta _2^ + } \over q}\right)\right)}\Gamma }} \right\rangle }^{{1 \over \Gamma }}}}}} \right)}}} \right]} \right)\cr =&  \left( {\left[ {{{\hat s}_{q\left( {1 - {e^{ - {{\left\langle {{{\left( - \log \left(1 - {{\theta _2^ - } \over q}\right)\right)}\Gamma } + {{\left( - \log \left(1 - {{\theta _1^ - } \over q}\right)\right)}\Gamma }} \right\rangle }^{{1 \over \Gamma }}}}}} \right)}},{{\hat s}_{q\left( {1 - {e^{ - {{\left\langle {{{\left( - \log \left(1 - {{\theta _2^ + } \over q}\right)\right)}\Gamma } + {{\left( - \log \left(1 - {{\theta _1^ + } \over q}\right)\right)}\Gamma }} \right\rangle }^{{1 \over \Gamma }}}}}} \right)}}} \right]} \right.,\cr & \left. {\left[ {{{\hat s}_{q\left( {{e^{ - {{\left\langle {{{\left( - \log \left({{\beta _2^ - } \over q}\right)\right)}\Gamma } + {{\left( - \log \left({{\beta _1^ - } \over q}\right)\right)}\Gamma }} \right\rangle }^{{1 \over \Gamma }}}}}} \right)}},{{\hat s}_{q\left( {{e^{ - {{\left\langle {{{\left( - \log \left({{\beta _2^ + } \over q}\right)\right)}\Gamma } + {{\left( - \log \left({{\beta _1^ + } \over q}\right)\right)}\Gamma }} \right\rangle }^{{1 \over \Gamma }}}}}} \right)}}} \right]} \right)\cr =& \, {Z_2} \oplus {Z_1}}$$

$${\eqalign{\left(ii\right) \,{Z_1} \otimes {Z_2}  =&  \left( {\left[ {{{\hat s}_{q\left( {{e^{ - {{\left\langle {{{\left( - \log \left({{\theta _1^ - } \over q}\right)\right)}\Gamma } + {{\left( - \log \left({{\theta _2^ - } \over q}\right)\right)}\Gamma }} \right\rangle }^{{1 \over \Gamma }}}}}} \right)}},{{\hat s}_{q\left( {{e^{ - {{\left\langle {{{\left( - \log \left({{\theta _1^ + } \over q}\right)\right)}\Gamma } + {{\left( - \log \left({{\theta _2^ + } \over q}\right)\right)}\Gamma }} \right\rangle }^{{1 \over \Gamma }}}}}} \right)}}} \right]} \right.,\cr & \left. {\left[ {{{\hat s}_{q\left( {1 - {e^{ - {{\left\langle {{{\left( - \log \left(1 - {{\beta _1^ - } \over q}\right)\right)}\Gamma } + {{\left( - \log \left(1 - {{\beta _2^ - } \over q}\right)\right)}\Gamma }} \right\rangle }^{{1 \over \Gamma }}}}}} \right)}},{{\hat s}_{q\left( {1 - {e^{ - {{\left\langle {{{\left( - \log \left(1 - {{\beta _1^ + } \over q}\right)\right)}\Gamma } + {{\left( - \log \left(1 - {{\beta _2^ + } \over q}\right)\right)}\Gamma }} \right\rangle }^{{1 \over \Gamma }}}}}} \right)}}} \right]} \right)\cr = &  \left( {\left[ {{{\hat s}_{q\left( {{e^{ - {{\left\langle {{{\left( - \log \left({{\theta _2^ - } \over q}\right)\right)}\Gamma } + {{\left( - \log \left({{\theta _1^ - } \over q}\right)\right)}\Gamma }} \right\rangle }^{{1 \over \Gamma }}}}}} \right)}},{{\hat s}_{q\left( {{e^{ - {{\left\langle {{{\left( - \log \left({{\theta _2^ + } \over q}\right)\right)}\Gamma } + {{\left( - \log \left({{\theta _1^ + } \over q}\right)\right)}\Gamma }} \right\rangle }^{{1 \over \Gamma }}}}}} \right)}}} \right]} \right.,\cr & \left. {\left[ {{{\hat s}_{q\left( {1 - {e^{ - {{\left\langle {{{\left( - \log \left(1 - {{\beta _2^ - } \over q}\right)\right)}\Gamma } + {{\left( - \log \left(1 - {{\beta _1^ - } \over q}\right)\right)}\Gamma }} \right\rangle }^{{1 \over \Gamma }}}}}} \right)}},{{\hat s}_{q\left( {1 - {e^{ - {{\left\langle {{{\left( - \log \left(1 - {{\beta _2^ + } \over q}\right)\right)}\Gamma } + {{\left( - \log \left(1 - {{\beta _1^ + } \over q}\right)\right)}\Gamma }} \right\rangle }^{{1 \over \Gamma }}}}}} \right)}}} \right]} \right)\cr=& \, {Z_2} \otimes {Z_1}}}$$

$${\eqalign{\left(iii\right)\,\alpha \left({Z_1} \oplus {Z_2}\right) =&  \left( {\left[ {{{\hat s}_{q\left( {1 - {e^{ - {{\left\langle {\alpha \left({{\left( - \log \left(1 - {{\theta _1^ - } \over q}\right)\right)}\Gamma } + {{\left( - \log \left(1 - {{\theta _2^ - } \over q}\right)\right)}\Gamma }\right)} \right\rangle }^{{1 \over \Gamma }}}}}} \right)}},{{\hat s}_{q\left( {1 - {e^{ - {{\left\langle {\alpha \left({{\left( - \log \left(1 - {{\theta _1^ + } \over q}\right)\right)}\Gamma } + {{\left( - \log \left(1 - {{\theta _2^ + } \over q}\right)\right)}\Gamma }\right)} \right\rangle }^{{1 \over \Gamma }}}}}} \right)}}} \right]} \right.,\cr & \left. {\left[ {{{\hat s}_{q\left( {{e^{ - {{\left\langle {\alpha \left({{\left( - \log \left({{\beta _1^ - } \over q}\right)\right)}\Gamma } + {{\left( - \log \left({{\beta _2^ - } \over q}\right)\right)}\Gamma }\right)} \right\rangle }^{{1 \over \Gamma }}}}}} \right)}},{{\hat s}_{q\left( {{e^{ - {{\left\langle {\alpha \left({{\left( - \log \left({{\beta _1^ + } \over q}\right)\right)}\Gamma } + {{\left( - \log \left({{\beta _2^ + } \over q}\right)\right)}\Gamma }\right)} \right\rangle }^{{1 \over \Gamma }}}}}} \right)}}} \right]} \right)\cr =&  \left( {\left[ {{{\hat s}_{q\left( {1 - {e^{ - {{\left\langle {\alpha {{\left( - \log \left(1 - {{\theta _1^ - } \over q}\right)\right)}\Gamma } + \alpha {{\left( - \log \left(1 - {{\theta _2^ - } \over q}\right)\right)}\Gamma }} \right\rangle }^{{1 \over \Gamma }}}}}} \right)}},{{\hat s}_{q\left( {1 - {e^{ - {{\left\langle {\alpha {{\left( - \log \left(1 - {{\theta _1^ + } \over q}\right)\right)}\Gamma } + \alpha {{\left( - \log \left(1 - {{\theta _2^ + } \over q}\right)\right)}\Gamma }} \right\rangle }^{{1 \over \Gamma }}}}}} \right)}}} \right]} \right.,\cr & \left. {\left[ {{{\hat s}_{q\left( {{e^{ - {{\left\langle {\alpha {{\left( - \log \left({{\beta _1^ - } \over q}\right)\right)}\Gamma } + \alpha {{\left( - \log \left({{\beta _2^ - } \over q}\right)\right)}\Gamma }} \right\rangle }^{{1 \over \Gamma }}}}}} \right)}},{{\hat s}_{q\left( {{e^{ - {{\left\langle {\alpha {{\left( - \log \left({{\beta _1^ + } \over q}\right)\right)}\Gamma } + \alpha {{\left( - \log \left({{\beta _2^ + } \over q}\right)\right)}\Gamma }} \right\rangle }^{{1 \over \Gamma }}}}}} \right)}}} \right]} \right)\cr =& \, \alpha {Z_1} \oplus \alpha {Z_2}}}$$

$${\eqalign{\left(iv\right)\,\left({\alpha _1} + {\alpha _2}\right){Z_1} =&  \left( {\left[ {{{\hat s}_{q\left( {1 - {e^{ - {{\left\langle {\left({\alpha _1} + {\alpha _2}\right){{\left( - \log \left(1 - {{\theta _1^ - } \over q}\right)\right)}\Gamma }} \right\rangle }^{{1 \over \Gamma }}}}}} \right)}},{{\hat s}_{q\left( {1 - {e^{ - {{\left\langle {\left({\alpha _1} + {\alpha _2}\right){{\left( - \log \left(1 - {{\theta _1^ + } \over q}\right)\right)}\Gamma }} \right\rangle }^{{1 \over \Gamma }}}}}} \right)}}} \right]} \right.,\cr & \left. {\left[ {{{\hat s}_{q\left( {{e^{ - {{\left\langle {\left({\alpha _1} + {\alpha _2}\right){{\left( - \log \left({{\beta _1^ - } \over q}\right)\right)}\Gamma }} \right\rangle }^{{1 \over \Gamma }}}}}} \right)}},{{\hat s}_{q\left( {{e^{ - {{\left\langle {\left({\alpha _1} + {\alpha _2}\right){{\left( - \log \left({{\beta _1^ + } \over q}\right)\right)}\Gamma }} \right\rangle }^{{1 \over \Gamma }}}}}} \right)}}} \right]} \right)\cr =&  \left( {\left[ {{{\hat s}_{q\left( {1 - {e^{ - {{\left\langle {{\alpha _1}{{\left( - \log \left(1 - {{\theta _1^ - } \over q}\right)\right)}\Gamma } + {\alpha _2}{{\left( - \log \left(1 - {{\theta _1^ - } \over q}\right)\right)}\Gamma }} \right\rangle }^{{1 \over \Gamma }}}}}} \right)}},{{\hat s}_{q\left( {1 - {e^{ - {{\left\langle {{\alpha _1}{{\left( - \log \left(1 - {{\theta _1^ + } \over q}\right)\right)}\Gamma } + {\alpha _2}{{\left( - \log \left(1 - {{\theta _1^ + } \over q}\right)\right)}\Gamma }} \right\rangle }^{{1 \over \Gamma }}}}}} \right)}}} \right]} \right.,\cr & \left. {\left[ {{{\hat s}_{q\left( {{e^{ - {{\left\langle {{\alpha _1}{{\left( - \log \left({{\beta _1^ - } \over q}\right)\right)}\Gamma } + {\alpha _2}{{\left( - \log \left({{\beta _1^ - } \over q}\right)\right)}\Gamma }} \right\rangle }^{{1 \over \Gamma }}}}}} \right)}},{{\hat s}_{q\left( {{e^{ - {{\left\langle {{\alpha _1}{{\left( - \log \left({{\beta _1^ + } \over q}\right)\right)}\Gamma } + {\alpha _2}{{\left( - \log \left({{\beta _1^ + } \over q}\right)\right)}\Gamma }} \right\rangle }^{{1 \over \Gamma }}}}}} \right)}}} \right]} \right)\cr =& \, {\alpha _1}{Z_1} + {\alpha _2}{Z_1}}}$$

$${\eqalign{\left(v\right)\,{\left({Z_1} \otimes {Z_2}\right)^\alpha } =&  \left( {\left[ {{{\hat s}_{q\left( {{e^{ - {{\left\langle {\alpha \left({{\left( - \log \left({{\theta _1^ - } \over q}\right)\right)}\Gamma } + {{\left( - \log \left({{\theta _2^ - } \over q}\right)\right)}\Gamma }\right)} \right\rangle }^{{1 \over \Gamma }}}}}} \right)}},{{\hat s}_{q\left( {{e^{ - {{\left\langle {\alpha \left({{\left( - \log \left({{\theta _1^ + } \over q}\right)\right)}\Gamma } + {{\left( - \log \left({{\theta _2^ + } \over q}\right)\right)}\Gamma }\right)} \right\rangle }^{{1 \over \Gamma }}}}}} \right)}}} \right]} \right.,\cr & \left. {\left[ {{{\hat s}_{q\left( {1 - {e^{ - {{\left\langle {\alpha \left({{\left( - \log \left(1 - {{\beta _1^ - } \over q}\right)\right)}\Gamma } + {{\left( - \log \left(1 - {{\beta _2^ - } \over q}\right)\right)}\Gamma }\right)} \right\rangle }^{{1 \over \Gamma }}}}}} \right)}},{{\hat s}_{q\left( {1 - {e^{ - {{\left\langle {\alpha \left({{\left( - \log \left(1 - {{\beta _1^ + } \over q}\right)\right)}\Gamma } + {{\left( - \log \left(1 - {{\beta _2^ + } \over q}\right)\right)}\Gamma }\right)} \right\rangle }^{{1 \over \Gamma }}}}}} \right)}}} \right]} \right)\cr =&  \left( {\left[ {{{\hat s}_{q\left( {{e^{ - {{\left\langle {\alpha {{\left( - \log \left({{\theta _1^ - } \over q}\right)\right)}\Gamma } + \alpha {{\left( - \log \left({{\theta _2^ - } \over q}\right)\right)}\Gamma }} \right\rangle }^{{1 \over \Gamma }}}}}} \right)}},{{\hat s}_{q\left( {{e^{ - {{\left\langle {\alpha {{\left( - \log \left({{\theta _1^ + } \over q}\right)\right)}\Gamma } + \alpha {{\left( - \log \left({{\theta _2^ + } \over q}\right)\right)}\Gamma }} \right\rangle }^{{1 \over \Gamma }}}}}} \right)}}} \right]} \right.,\cr &  \left. {\left[ {{{\hat s}_{q\left( {1 - {e^{ - {{\left\langle {\alpha {{\left( - \log \left(1 - {{\beta _1^ - } \over q}\right)\right)}\Gamma } + \alpha {{\left( - \log \left(1 - {{\beta _2^ - } \over q}\right)\right)}\Gamma }} \right\rangle }^{{1 \over \Gamma }}}}}} \right)}},{{\hat s}_{q\left( {1 - {e^{ - {{\left\langle {\alpha {{\left( - \log \left(1 - {{\beta _1^ + } \over q}\right)\right)}\Gamma } + \alpha {{\left( - \log \left(1 - {{\beta _2^ + } \over q}\right)\right)}\Gamma }} \right\rangle }^{{1 \over \Gamma }}}}}} \right)}}} \right]} \right)\cr =& \, Z_{1}^{\alpha}\otimes Z_{2}^{\alpha}}}$$

$$\eqalign{\left(vi\right)\, Z_1^{{\alpha _1}} \otimes Z_1^{{\alpha _2}} =&  \left( {\left[ {{{\hat s}_{q\left( {{e^{ - {{\left\langle {{\alpha _1}{{\left( - \log \left({{\theta _1^ - } \over q}\right)\right)}\Gamma } + {\alpha _2}{{\left( - \log \left({{\theta _1^ - } \over q}\right)\right)}\Gamma }} \right\rangle }^{{1 \over \Gamma }}}}}} \right)}},{{\hat s}_{q\left( {{e^{ - {{\left\langle {{\alpha _1}{{\left( - \log \left({{\theta _1^ + } \over q}\right)\right)}\Gamma } + {\alpha _2}{{\left( - \log \left({{\theta _1^ + } \over q}\right)\right)}\Gamma }} \right\rangle }^{{1 \over \Gamma }}}}}} \right)}}} \right]} \right.,\cr &  \left. {\left[ {{{\hat s}_{q\left( {1 - {e^{ - {{\left\langle {{\alpha _1}{{\left( - \log \left(1 - {{\beta _1^ - } \over q}\right)\right)}\Gamma } + {\alpha _2}{{\left( - \log \left(1 - {{\beta _1^ - } \over q}\right)\right)}\Gamma }} \right\rangle }^{{1 \over \Gamma }}}}}} \right)}},{{\hat s}_{q\left( {1 - {e^{ - {{\left\langle {{\alpha _1}{{\left( - \log \left(1 - {{\beta _1^ + } \over q}\right)\right)}\Gamma } + {\alpha _2}{{\left( - \log \left(1 - {{\beta _1^ + } \over q}\right)\right)}\Gamma }} \right\rangle }^{{1 \over \Gamma }}}}}} \right)}}} \right]} \right)\cr =&  \left( {\left[ {{{\hat s}_{q\left( {{e^{ - {{\left\langle {\left({\alpha _1} + {\alpha _2}\right){{\left( - \log \left({{\theta _1^ - } \over q}\right)\right)}\Gamma }} \right\rangle }^{{1 \over \Gamma }}}}}} \right)}},{{\hat s}_{q\left( {{e^{ - {{\left\langle {\left({\alpha _1} + {\alpha _2}\right){{\left( - \log \left({{\theta _1^ + } \over q}\right)\right)}\Gamma }} \right\rangle }^{{1 \over \Gamma }}}}}} \right)}}} \right]} \right.,\cr &  \left. {\left[ {{{\hat s}_{q\left( {1 - {e^{ - {{\left\langle {\left({\alpha _1} + {\alpha _2}\right){{\left( - \log \left(1 - {{\beta _1^ - } \over q}\right)\right)}\Gamma }} \right\rangle }^{{1 \over \Gamma }}}}}} \right)}},{{\hat s}_{q\left( {1 - {e^{ - {{\left\langle {\left({\alpha _1} + {\alpha _2}\right){{\left( - \log \left(1 - {{\beta _1^ + } \over q}\right)\right)}\Gamma }} \right\rangle }^{{1 \over \Gamma }}}}}} \right)}}} \right]} \right)\cr =& \, Z_1^{\left({\alpha _1} + {\alpha _2}\right)}.}$$

## Livif aczel-alsina average aggregation operators

This section contains operators for the Aczel-Alsina average aggregates, such as LIVIFAAWA, LIVIFAAOWA, and LIVIFAAHA operators.

**Definition 8** *Assume that* ${Z_j} = \left([\hat s_{{\theta _j}}^ - ,\hat s_{{\theta _j}}^ + ],[\hat s_{{\beta _j}}^ - ,\hat s_{{\beta _j}}^ + ]\right)$ *is LIVIFNs. The LIVIFAAWA operator is* $LIVIFAAWA:LIVIF{N^k} \to LIVIFN$ *defined as:*

$$LIVIFAAWA\left({Z_1},{Z_2}, \ldots ,{Z_k}\right) =\mathop \oplus \limits_{j = 1}^k\left({\lambda _j}{Z_j}\right) = {\lambda _1}{Z_1} \oplus {\lambda _2}{Z_2} \oplus \cdots \oplus {\lambda _k}{Z_k},$$

*where* $\lambda = {\left({\lambda _1},{\lambda _2}, \ldots ,{\lambda _k}\right)^T}$ *and* $\sum\nolimits_{j = 1}^k {{\lambda _j}} = 1$.

We have the following theorem based on Aczel-Alsina operations using LIVIFNs.

**Theorem 2** *Assume that* ${Z_j} = \left([\hat s_{{\theta _j}}^ - ,\hat s_{{\theta _j}}^ + ],[\hat s_{{\beta _j}}^ - ,\hat s_{{\beta _j}}^ + ]\right)$ *is LIVIFNs. Then*

(2) $$\eqalign{ & LIVIFAAWA\left({Z_1},{Z_2}, \ldots ,{Z_k}\right)\cr = &  \left( {\matrix{ {\left[ {{{\hat s}_{q\left( {1 - {e^{ - {{\left\langle {\sum\limits_{j = 1}^k {{\lambda _j}} {{\left( - \log \left(1 - {{\theta _j^ - } \over q}\right)\right)}\Gamma }} \right\rangle }^{{1 \over \Gamma }}}}}} \right)}},{{\hat s}_{q\left( {1 - {e^{ - {{\left\langle {\sum\limits_{j = 1}^k {{\lambda _j}} {{\left( - \log \left(1 - {{\theta _j^ + } \over q}\right)\right)}\Gamma }} \right\rangle }^{{1 \over \Gamma }}}}}} \right)}}} \right],} \cr {\left[ {{{\hat s}_{q\left( {{e^{ - {{\left\langle {\sum\limits_{j = 1}^k {{\lambda _j}} {{\left( - \log \left({{\beta _j^ - } \over q}\right)\right)}\Gamma }} \right\rangle }^{{1 \over \Gamma }}}}}} \right)}},{{\hat s}_{q\left( {{e^{ - {{\left\langle {\sum\limits_{j = 1}^k {{\lambda _j}} {{\left( - \log \left({{\beta _j^ + } \over q}\right)\right)}\Gamma }} \right\rangle }^{{1 \over \Gamma }}}}}} \right)}}} \right]} } } \right)}.$$

*Here* $\lambda = {\left({\lambda _1},{\lambda _2}, \ldots ,{\lambda _k}\right)^T}$.

**Proof:** We employ the mathematical induction method to demonstrate this theorem.

(i) Let $k = 2,$ then using Aczel-Alsina operations of LIVIFNs, we obtain

$${\lambda _1}{Z_1} = \left( {\matrix{ {\left[ {{{\hat s}_{q\left( {1 - {e^{ - {{\left\langle {{\lambda _1}{{\left( - \log \left(1 - {{\theta _1^ - } \over q}\right)\right)}\Gamma }} \right\rangle }^{{1 \over \Gamma }}}}}} \right)}},{{\hat s}_{q\left( {1 - {e^{ - {{\left\langle {{\lambda _1}{{\left( - \log \left(1 - {{\theta _1^ + } \over q}\right)\right)}\Gamma }} \right\rangle }^{{1 \over \Gamma }}}}}} \right)}}} \right],} \cr  {\left[ {{{\hat s}_{q\left( {{e^{ - {{\left\langle {{\lambda _1}{{\left( - \log \left({{\beta _1^ - } \over q}\right)\right)}\Gamma }} \right\rangle }^{{1 \over \Gamma }}}}}} \right)}},{{\hat s}_{q\left( {{e^{ - {{\left\langle {{\lambda _1}{{\left( - \log \left({{\beta _1^ + } \over q}\right)\right)}\Gamma }} \right\rangle }^{{1 \over \Gamma }}}}}} \right)}}} \right]} \cr  } } \right)$$

$${{\lambda _2}{Z_2} = \left( {\matrix{ {\left[ {{{\hat s}_{q\left( {1 - {e^{ - {{\left\langle {{\lambda _2}{{\left( - \log \left(1 - {{\theta _2^ - } \over q}\right)\right)}\Gamma }} \right\rangle }^{{1 \over \Gamma }}}}}} \right)}},{{\hat s}_{q\left( {1 - {e^{ - {{\left\langle {{\lambda _2}{{\left( - \log \left(1 - {{\theta _2^ + } \over q}\right)\right)}\Gamma }} \right\rangle }^{{1 \over \Gamma }}}}}} \right)}}} \right],} \cr  {\left[ {{{\hat s}_{q\left( {{e^{ - {{\left\langle {{\lambda _2}{{\left( - \log \left({{\beta _2^ - } \over q}\right)\right)}\Gamma }} \right\rangle }^{{1 \over \Gamma }}}}}} \right)}},{{\hat s}_{q\left( {{e^{ - {{\left\langle {{\lambda _2}{{\left( - \log \left({{\beta _2^ + } \over q}\right)\right)}\Gamma }} \right\rangle }^{{1 \over \Gamma }}}}}} \right)}}} \right]} \cr  } } \right)}.$$

Using Definition 7, we get

$$\eqalign{& LIVIFAAWA\left({Z_1},{Z_2}\right) = {\lambda _1}{Z_1}\mathop \oplus \limits_2 {\lambda _2}{Z_2}\cr =&  \left( {\matrix{ {\left[ {{{\hat s}_{q\left( {1 - {e^{ - {{\left\langle {{\lambda _1}{{\left( - \log \left(1 - {{\theta _1^ - } \over q}\right)\right)}\Gamma }} \right\rangle }^{{1 \over \Gamma }}}}}} \right)}},{{\hat s}_{q\left( {1 - {e^{ - {{\left\langle {{\lambda _1}{{\left( - \log \left(1 - {{\theta _1^ + } \over q}\right)\right)}\Gamma }} \right\rangle }^{{1 \over \Gamma }}}}}} \right)}}} \right],} \cr {\left[ {{{\hat s}_{q\left( {{e^{ - {{\left\langle {{\lambda _1}{{\left( - \log \left({{\beta _1^ - } \over q}\right)\right)}\Gamma }} \right\rangle }^{{1 \over \Gamma }}}}}} \right)}},{{\hat s}_{q\left( {{e^{ - {{\left\langle {{\lambda _1}{{\left( - \log \left({{\beta _1^ + } \over q}\right)\right)}\Gamma }} \right\rangle }^{{1 \over \Gamma }}}}}} \right)}}} \right]}} } \right)\cr &  \oplus \left( {\matrix{ {\left[ {{{\hat s}_{q\left( {1 - {e^{ - {{\left\langle {{\lambda _2}{{\left( - \log \left(1 - {{\theta _2^ - } \over q}\right)\right)}\Gamma }} \right\rangle }^{{1 \over \Gamma }}}}}} \right)}},{{\hat s}_{q\left( {1 - {e^{ - {{\left\langle {{\lambda _2}{{\left( - \log \left(1 - {{\theta _2^ + } \over q}\right)\right)}\Gamma }} \right\rangle }^{{1 \over \Gamma }}}}}} \right)}}} \right],} \cr {\left[ {{{\hat s}_{q\left( {{e^{ - {{\left\langle {{\lambda _2}{{\left( - \log \left({{\beta _2^ - } \over q}\right)\right)}\Gamma }} \right\rangle }^{{1 \over \Gamma }}}}}} \right)}},{{\hat s}_{q\left( {{e^{ - {{\left\langle {{\lambda _2}{{\left( - \log \left({{\beta _2^ + } \over q}\right)\right)}\Gamma }} \right\rangle }^{{1 \over \Gamma }}}}}} \right)}}} \right]}} } \right)}$$

$$\eqalign{ = \left( {\matrix{ {\left[ {{{\hat s}_{q\left( {1 - {e^{ - {{\left\langle {{\lambda _1}{{\left( - \log \left(1 - {{\theta _1^ - } \over q}\right)\right)}\Gamma } + {\lambda _2}{{\left( - \log \left(1 - {{\theta _2^ - } \over q}\right)\right)}\Gamma }} \right\rangle }^{{1 \over \Gamma }}}}}} \right)}},{{\hat s}_{q\left( {1 - {e^{ - {{\left\langle {{\lambda _1}{{\left( - \log \left(1 - {{\theta _1^ + } \over q}\right)\right)}\Gamma } + {\lambda _2}{{\left( - \log \left(1 - {{\theta _2^ + } \over q}\right)\right)}\Gamma }} \right\rangle }^{{1 \over \Gamma }}}}}} \right)}}} \right],} \cr {\left[ {{{\hat s}_{q\left( {{e^{ - {{\left\langle {{\lambda _1}{{\left( - \log \left({{\beta _1^ - } \over q}\right)\right)}\Gamma } + {\lambda _2}{{\left( - \log \left({{\beta _2^ - } \over q}\right)\right)}\Gamma }} \right\rangle }^{{1 \over \Gamma }}}}}} \right)}},{{\hat s}_{q\left( {{e^{ - {{\left\langle {{\lambda _1}{{\left( - \log \left({{\beta _1^ + } \over q}\right)\right)}\Gamma } + {\lambda _2}{{\left( - \log \left({{\beta _2^ + } \over q}\right)\right)}\Gamma }} \right\rangle }^{{1 \over \Gamma }}}}}} \right)}}} \right]} \cr } } \right)\hfill\\{{ = \left( {\matrix{ {\left[ {{{\hat s}_{q\left( {1 - {e^{ - {{\left\langle {\sum\limits_{j = 1}^2 {{\lambda _j}} {{\left( - \log \left(1 - {{\theta _j^ - } \over q}\right)\right)}\Gamma }} \right\rangle }^{{1 \over \Gamma }}}}}} \right)}},{{\hat s}_{q\left( {1 - {e^{ - {{\left\langle {\sum\limits_{j = 1}^2 {{\lambda _j}} {{\left( - \log \left(1 - {{\theta _j^ + } \over q}\right)\right)}\Gamma }} \right\rangle }^{{1 \over \Gamma }}}}}} \right)}}} \right],} \cr {\left[ {{{\hat s}_{q\left( {{e^{ - {{\left\langle {\sum\limits_{j = 1}^2 {{\lambda _j}} {{\left( - \log \left({{\beta _j^ - } \over q}\right)\right)}\Gamma }} \right\rangle }^{{1 \over \Gamma }}}}}} \right)}},{{\hat s}_{q\left( {{e^{ - {{\left\langle {\sum\limits_{j = 1}^2 {{\lambda _j}} {{\left( - \log \left({{\beta _j^ + } \over q}\right)\right)}\Gamma }} \right\rangle }^{{1 \over \Gamma }}}}}} \right)}}} \right]}} } \right)}.}\hfill}$$

Hence, Eq. (2) satisfies for $k = 2$.

(ii) Suppose that Eq. (2) is true for $k = m$, *i.e.*,

$$\eqalign{& LIVIFAAWA\left({Z_1},{Z_2}, \ldots ,{Z_m}\right) = \mathop \oplus \limits _{j = 1}^m\left({\lambda _j}{Z_j}\right)\cr = &  \left( {\matrix{ {\left[ {{{\hat s}_{q\left( {1 - {e^{ - {{\left\langle {\sum\limits_{j = 1}^m {{\lambda _j}} {{\left( - \log \left(1 - {{\theta _j^ - } \over q}\right)\right)}\Gamma }} \right\rangle }^{{1 \over \Gamma }}}}}} \right)}},{{\hat s}_{q\left( {1 - {e^{ - {{\left\langle {\sum\limits_{j = 1}^m {{\lambda _j}} {{\left( - \log \left(1 - {{\theta _j^ + } \over q}\right)\right)}\Gamma }} \right\rangle }^{{1 \over \Gamma }}}}}} \right)}}} \right],} \cr {\left[ {{{\hat s}_{q\left( {{e^{ - {{\left\langle {\sum\limits_{j = 1}^m {{\lambda _j}} {{\left( - \log \left({{\beta _j^ - } \over q}\right)\right)}\Gamma }} \right\rangle }^{{1 \over \Gamma }}}}}} \right)}},{{\hat s}_{q\left( {{e^{ - {{\left\langle {\sum\limits_{j = 1}^m {{\lambda _j}} {{\left( - \log \left({{\beta _j^ + } \over q}\right)\right)}\Gamma }} \right\rangle }^{{1 \over \Gamma }}}}}} \right)}}} \right]}} } \right)}.$$

(iii) Now consider $m = k + 1$, then we have

$$\eqalign{&  LIVIFAAWA\left({Z_1},{Z_2}, \ldots ,{Z_{m + 1}}\right) = \mathop \oplus \limits _{j = 1}^m\left({\lambda _j}{Z_j}\right) \oplus \left({\lambda _{m + 1}}{Z_{m + 1}}\right)\cr =&  \left( {\matrix{ {\left[ {{{\hat s}_{q\left( {1 - {e^{ - {{\left\langle {\sum\limits_{j = 1}^m {{\lambda _j}} {{\left( - \log \left(1 - {{\theta _j^ - } \over q}\right)\right)}\Gamma }} \right\rangle }^{{1 \over \Gamma }}}}}} \right)}},{{\hat s}_{q\left( {1 - {e^{ - {{\left\langle {\sum\limits_{j = 1}^m {{\lambda _j}} {{\left( - \log \left(1 - {{\theta _j^ + } \over q}\right)\right)}\Gamma }} \right\rangle }^{{1 \over \Gamma }}}}}} \right)}}} \right],} \cr {\left[ {{{\hat s}_{q\left( {{e^{ - {{\left\langle {\sum\limits_{j = 1}^m {{\lambda _j}} {{\left( - \log \left({{\beta _j^ - } \over q}\right)\right)}\Gamma }} \right\rangle }^{{1 \over \Gamma }}}}}} \right)}},{{\hat s}_{q\left( {{e^{ - {{\left\langle {\sum\limits_{j = 1}^m {{\lambda _j}} {{\left( - \log \left({{\beta _j^ + } \over q}\right)\right)}\Gamma }} \right\rangle }^{{1 \over \Gamma }}}}}} \right)}}} \right]} \cr } } \right)\cr &  \oplus \left( {\matrix{ {\left[ {{{\hat s}_{q\left( {1 - {e^{ - {{\left\langle {{\lambda _{m + 1}}{{\left( - \log \left(1 - {{\theta _{m + 1}^ - } \over q}\right)\right)}\Gamma }} \right\rangle }^{{1 \over \Gamma }}}}}} \right)}},{{\hat s}_{q\left( {1 - {e^{ - {{\left\langle {{\lambda _{m + 1}}{{\left( - \log \left(1 - {{\theta _{m + 1}^ + } \over q}\right)\right)}\Gamma }} \right\rangle }^{{1 \over \Gamma }}}}}} \right)}}} \right],} \cr {\left[ {{{\hat s}_{q\left( {{e^{ - {{\left\langle {{\lambda _{m + 1}}{{\left( - \log \left({{\beta _{m + 1}^ - } \over q}\right)\right)}\Gamma }} \right\rangle }^{{1 \over \Gamma }}}}}} \right)}},{{\hat s}_{q\left( {{e^{ - {{\left\langle {{\lambda _{m + 1}}{{\left( - \log \left({{\beta _{m + 1}^ + } \over q}\right)\right)}\Gamma }} \right\rangle }^{{1 \over \Gamma }}}}}} \right)}}} \right]} \cr } } \right)\cr =&  \left( {\matrix{ {\left[ {{{\hat s}_{q\left( {1 - {e^{ - {{\left\langle {\sum\limits_{j = 1}^{m + 1} {{\lambda _j}} {{\left( - \log \left(1 - {{\theta _j^ - } \over q}\right)\right)}\Gamma }} \right\rangle }^{{1 \over \Gamma }}}}}} \right)}},{{\hat s}_{q\left( {1 - {e^{ - {{\left\langle {\sum\limits_{j = 1}^{m + 1} {{\lambda _j}} {{\left( - \log \left(1 - {{\theta _j^ + } \over q}\right)\right)}\Gamma }} \right\rangle }^{{1 \over \Gamma }}}}}} \right)}}} \right],} \cr {\left[ {{{\hat s}_{q\left( {{e^{ - {{\left\langle {\sum\limits_{j = 1}^{m + 1} {{\lambda _j}} {{\left( - \log \left({{\beta _j^ - } \over q}\right)\right)}\Gamma }} \right\rangle }^{{1 \over \Gamma }}}}}} \right)}},{{\hat s}_{q\left( {{e^{ - {{\left\langle {\sum\limits_{j = 1}^{m + 1} {{\lambda _j}} {{\left( - \log \left({{\beta _j^ + } \over q}\right)\right)}\Gamma }} \right\rangle }^{{1 \over \Gamma }}}}}} \right)}}} \right]}} } \right)}.$$

Thus, Eq. (2) is true for $k = m + 1$. Hence, Eq. (2) satisfies any value of $k$.

The following are the properties which can easily be proved using the LIVIFAAWA operator.

**Theorem 3** *(Idempotency property) Let* ${Z_j} = \left([\hat s_{{\theta _j}}^ - ,\hat s_{{\theta _j}}^ + ],[\hat s_{{\beta _j}}^ - ,\hat s_{{\beta _j}}^ + ]\right)$ *be LIVIFNs. If for each* $j,$ ${Z_j} = {Z_1}$, *then* $LIVIFAAWA\left({Z_1},{Z_2}, \ldots ,{Z_k}\right) = Z$.

**Proof:** Given that ${Z_j} = \left([\hat s_{{\theta _j}}^ - ,\hat s_{{\theta _j}}^ + ],[\hat s_{{\beta _j}}^ - ,\hat s_{{\beta _j}}^ + ]\right) = {Z_1}$ for each, then from Eq. (2), we have

$$\eqalign{&  LIVIFAAWA\left({Z_1},{Z_2}, \ldots ,{Z_k}\right) = \mathop \oplus \limits _{j = 1}^k\left({\lambda _j}{Z_j}\right)\cr &  = \left( {\matrix{ {\left[ {{{\hat s}_{q\left( {1 - {e^{ - {{\left\langle {\sum\limits_{j = 1}^k {{\lambda _j}} {{\left( - \log \left(1 - {{\theta _j^ - } \over q}\right)\right)}\Gamma }} \right\rangle }^{{1 \over \Gamma }}}}}} \right)}},{{\hat s}_{q\left( {1 - {e^{ - {{\left\langle {\sum\limits_{j = 1}^k {{\lambda _j}} {{\left( - \log \left(1 - {{\theta _j^ + } \over q}\right)\right)}\Gamma }} \right\rangle }^{{1 \over \Gamma }}}}}} \right)}}} \right],} \cr {\left[ {{{\hat s}_{q\left( {{e^{ - {{\left\langle {\sum\limits_{j = 1}^k {{\lambda _j}} {{\left( - \log \left({{\beta _j^ - } \over q}\right)\right)}\Gamma }} \right\rangle }^{{1 \over \Gamma }}}}}} \right)}},{{\hat s}_{q\left( {{e^{ - {{\left\langle {\sum\limits_{j = 1}^k {{\lambda _j}} {{\left( - \log \left({{\beta _j^ + } \over q}\right)\right)}\Gamma }} \right\rangle }^{{1 \over \Gamma }}}}}} \right)}}} \right]} \cr } } \right)\cr &  = \left( {\matrix{ {\left[ {{{\hat s}_{q\left( {1 - {e^{ - {{\left\langle {{{\left( - \log \left(1 - {{\theta _1^ - } \over q}\right)\right)}\Gamma }} \right\rangle }^{{1 \over \Gamma }}}}}} \right)}},{{\hat s}_{q\left( {1 - {e^{ - {{\left\langle {{{\left( - \log \left(1 - {{\theta _1^ + } \over q}\right)\right)}\Gamma }} \right\rangle }^{{1 \over \Gamma }}}}}} \right)}}} \right],} \cr {\left[ {{{\hat s}_{q\left( {{e^{ - {{\left\langle {{{\left( - \log \left({{\beta _1^ - } \over q}\right)\right)}\Gamma }} \right\rangle }^{{1 \over \Gamma }}}}}} \right)}},{{\hat s}_{q\left( {{e^{ - {{\left\langle {{{\left( - \log \left({{\beta _1^ + } \over q}\right)\right)}\Gamma }} \right\rangle }^{{1 \over \Gamma }}}}}} \right)}}} \right]} \cr } } \right)}$$

$$\eqalign{{ =   \left({\matrix{ {\left[ {{{\hat s}_{q\left( {1 - {e^{ - \left( - \log \left(1 - {{\theta _1^ - } \over q}\right)\right)}}} \right)}},{{\hat s}_{q\left( {1 - {e^{ - \left( - \log \left(1 - {{\theta _1^ + } \over q}\right)\right)}}} \right)}}} \right],} \cr {\left[ {{{\hat s}_{q\left( {{e^{ - \left( - \log \left({{\beta _1^ - } \over q}\right)\right)}}} \right)}},{{\hat s}_{q\left( {{e^{ - \left( - \log \left({{\beta _1^ + } \over q}\right)\right)}}} \right)}}} \right]} } } \right)}}$$

$$\eqalign{& {= \left( {\matrix{ {\left[ {{{\hat s}_{q\left( {1 - {e^{\log \left(1 - {{\theta _1^ - } \over q}\right)}}} \right)}},{{\hat s}_{q\left( {1 - {e^{\log \left(1 - {{\theta _1^ + } \over q}\right)}}} \right)}}} \right],} \cr {\left[ {{{\hat s}_{q\left( {{e^{\log \left({{\beta _1^ - } \over q}\right)}}} \right)}},{{\hat s}_{q\left( {{e^{\log \left({{\beta _1^ + } \over q}\right)}}} \right)}}} \right]} \cr } } \right)}\cr &  {= \left([\hat s_{{\theta _1}}^{-} ,\hat s_{{\theta _1}}^ + ],[\hat s_{{\beta _1}}^{-} ,\hat s_{{\beta _1}}^ + ]\right) = {Z_1}.}}$$

Hence, $LIVIFAAWA\left({Z_1},{Z_2}, \ldots ,{Z_m}\right) = {Z_1}$.

**Theorem 4** *(Boundedness property) Let* ${Z_j} = \left([\hat s_{{\theta _j}}^ - ,\hat s_{{\theta _j}}^ + ],[\hat s_{{\beta _j}}^ - ,\hat s_{{\beta _j}}^ + ]\right)$. *If* ${Z^ * } = \left([\hat s_{{\theta ^ * }}^ - ,\hat s_{{\theta ^ * }}^ + ],[\hat s_{{\beta ^ * }}^ - ,\right.$ $\hat s_{{\beta ^ * }}^ + ])$ *such that* $\hat s_{{\theta ^ * }}^ - = \mathop {\min }\limits_j \{ \hat s_{{\theta _j}}^ - \} ,\hat s_{{\theta ^ * }}^ + = \mathop {\min }\limits_j \{ \hat s_{{\theta _j}}^ + \}$, $\hat s_{{\beta ^ * }}^ - = \mathop {\max }\limits_j \{ \hat s_{{\beta _j}}^ - \} ,$ $\hat s_{{\beta ^ * }}^ + = \mathop {\max }\limits_j \{ \hat s_{{\beta _j}}^ + \}$ *and* ${Z^{ * * }} = \left([\hat s_{{\theta ^{ * * }}}^ - ,\hat s_{{\theta ^{ * * }}}^ + ],[\hat s_{{\beta ^{ * * }}}^ - ,\hat s_{{\beta ^{ * * }}}^ + ]\right)$ *where* $\hat s_{{\theta ^{ * * }}}^ - = \mathop {\max }\limits_j \{ \hat s_{{\theta _j}}^ - \} ,$ $\hat s_{{\theta ^{ * * }}}^ + = \mathop {\max }\limits_j \{ \hat s_{{\theta _j}}^ + \}$, $\hat s_{{\beta ^{ * * }}}^ - = \mathop {\min }\limits_j \{ \hat s_{{\beta _j}}^ - \} ,$ $\hat s_{{\beta ^{ * * }}}^ + = \mathop {\min }\limits_j \{ \hat s_{{\beta _j}}^ + \} .$ *Then* ${Z^ * } \le LIVIFAAWA({Z_1},$ ${Z_2}, \ldots ,{Z_k}) \le {Z^{ * * }}.$

**Proof:** According to the hypothesis, we have the subsequent inequalities,

$$\eqalign{{\hat s_{q\left( {1 - {e^{ - {{\left\langle {\sum\limits_{j = 1}^k {{\lambda _j}} {{\left( - \log \left(1 - {{\theta _j^{ * - }} \over q}\right)\right)}\Gamma }} \right\rangle }^{{1 \over \Gamma }}}}}} \right)}}&  \le {\hat s_{q\left( {1 - {e^{ - {{\left\langle {\sum\limits_{j = 1}^k {{\lambda _j}} {{\left( - \log \left(1 - {{\theta _j^ - } \over q}\right)\right)}\Gamma }} \right\rangle }^{{1 \over \Gamma }}}}}} \right)}}\cr &  \le {\hat s_{q\left( {1 - {e^{ - {{\left\langle {\sum\limits_{j = 1}^k {{\lambda _j}} {{\left( - \log \left(1 - {{\theta _j^{ * * - }} \over q}\right)\right)}\Gamma }} \right\rangle }^{{1 \over \Gamma }}}}}} \right)}}, \cr {\hat s_{q\left( {1 - {e^{ - {{\left\langle {\sum\limits_{j = 1}^k {{\lambda _j}} {{\left( - \log \left(1 - {{\theta _j^{ * + }} \over q}\right)\right)}\Gamma }} \right\rangle }^{{1 \over \Gamma }}}}}} \right)}} &  \le {\hat s_{q\left( {1 - {e^{ - {{\left\langle {\sum\limits_{j = 1}^k {{\lambda _j}} {{\left( - \log \left(1 - {{\theta _j^ + } \over q}\right)\right)}\Gamma }} \right\rangle }^{{1 \over \Gamma }}}}}} \right)}}\cr &  \le {\hat s_{q\left( {1 - {e^{ - {{\left\langle {\sum\limits_{j = 1}^k {{\lambda _j}} {{\left( - \log \left(1 - {{\theta _j^{ * * + }} \over q}\right)\right)}\Gamma }} \right\rangle }^{{1 \over \Gamma }}}}}} \right)}} \cr  {\hat s_{q\left( {{e^{ - {{\left\langle {\sum\limits_{j = 1}^k {{\lambda _j}} {{\left( - \log \left({{\beta _j^{ * * - }} \over q}\right)\right)}\Gamma }} \right\rangle }^{{1 \over \Gamma }}}}}} \right)}}&  \le {\hat s_{q\left( {{e^{ - {{\left\langle {\sum\limits_{j = 1}^k {{\lambda _j}} {{\left( - \log \left({{\beta _j^ - } \over q}\right)\right)}\Gamma }} \right\rangle }^{{1 \over \Gamma }}}}}} \right)}}\cr &  \le {\hat s_{q\left( {{e^{ - {{\left\langle {\sum\limits_{j = 1}^k {{\lambda _j}} {{\left( - \log \left({{\beta _j^{ * - }} \over q}\right)\right)}\Gamma }} \right\rangle }^{{1 \over \Gamma }}}}}} \right)}}\cr   {\hat s_{q\left( {{e^{ - {{\left\langle {\sum\limits_{j = 1}^k {{\lambda _j}} {{\left( - \log \left({{\beta _j^{ * * + }} \over q}\right)\right)}\Gamma }} \right\rangle }^{{1 \over \Gamma }}}}}} \right)}}&  \le {\hat s_{q\left( {{e^{ - {{\left\langle {\sum\limits_{j = 1}^k {{\lambda _j}} {{\left( - \log \left({{\beta _j^ + } \over q}\right)\right)}\Gamma }} \right\rangle }^{{1 \over \Gamma }}}}}} \right).}}\cr &  \le {\hat s_{q\left( {{e^{ - {{\left\langle {\sum\limits_{j = 1}^k {{\lambda _j}} {{\left( - \log \left({{\beta _j^{ * + }} \over q}\right)\right)}\Gamma }} \right\rangle }^{{1 \over \Gamma }}}}}} \right)}}}$$

Hence, ${Z^ * } \le LIVIFAAWA\left({Z_1},{Z_2}, \ldots ,{Z_k}\right) \le$ ${Z^{ * * }}.$

**Theorem 5** *(Monotonicity property) Let* ${Z_j}$ *and* $\acute{Z}_j$ $\left(j = 1,2, \ldots ,k\right)$ *be two LIVIF sets, if* ${Z_j} \le \acute{Z}_j$ *for all* $j$, *then* $LIVIFAAWA\left({Z_1},{Z_2}, \ldots ,{Z_k}\right) \le LIVIFAAWA\left(\acute{Z}_1,\acute{Z}_2, \ldots ,\acute{Z}_k\right).$

Now, we define LIVIFAAOWA operator.

**Definition 9** *Assume that* ${Z_j} = \left([\hat s_{{\theta _j}}^ - ,\hat s_{{\theta _j}}^ + ],[\hat s_{{\beta _j}}^ - ,\hat s_{{\beta _j}}^ + ]\right)$ *Then LIVIFAAOWA operator mapping* $LIVIFAAOWA:LIVIF{N^k} \to LIVIFN$ *having weight vector* $\lambda = {\left({\lambda _1},{\lambda _2}, \ldots ,{\lambda _k}\right)^T}$ *with* ${\lambda _j} \in \left[ {0,1} \right]$ *and* $\sum\limits_{j = 1}^k {{\lambda _j}} = 1$, *i.e*., $LIVIFAAOWA\left({Z_1},{Z_2}, \ldots ,{Z_k}\right) = \mathop \oplus \limits _{j = 1}^k\left({\lambda _j}{Z_{P\left(j\right)}}\right).$

**Theorem 6** *Let* ${Z_j} = \left([\hat s_{{\theta _j}}^ - ,\hat s_{{\theta _j}}^ + ],[\hat s_{{\beta _j}}^ - ,\hat s_{{\beta _j}}^ + ]\right)$ $\left(j = 1,2, \ldots ,k\right)$ *be a set of LIVIFNs. Then LIVIF Aczel-Alsina ordered weighted average (LIVIFAAOWA) operator is a mapping* $LIVIFAAOWA:LIVIF{N^k} \to LIVIFN$ *with a concern weight vector* $\lambda = {\left({\lambda _1},{\lambda _2}, \ldots ,{\lambda _k}\right)^T}$, *also* ${\lambda _j} \in \left[ {0,1} \right]$ *and* $\sum\nolimits_{j = 1}^k {{\lambda _j}} = 1$, *i.e.*,

$$\eqalign{&  LIVIFAAOWA\left({Z_1},{Z_2}, \ldots ,{Z_k}\right) = \mathop \oplus \limits _{j = 1}^k\left({\lambda _j}{Z_{P\left(j\right)}}\right)\cr = &  \left( {\matrix{ {\left[ {{{\hat s}_{q\left( {1 - {e^{ - {{\left\langle {\sum\limits_{j = 1}^k {{\lambda _j}} {{\left( - \log \left(1 - {{\theta _{P\left(j\right)}^ - } \over q}\right)\right)}\Gamma }} \right\rangle }^{{1 \over \Gamma }}}}}} \right)}},{{\hat s}_{q\left( {1 - {e^{ - {{\left\langle {\sum\limits_{j = 1}^k {{\lambda _j}} {{\left( - \log \left(1 - {{\theta _{P\left(j\right)}^ + } \over q}\right)\right)}\Gamma }} \right\rangle }^{{1 \over \Gamma }}}}}} \right)}}} \right],} \cr {\left[ {{{\hat s}_{q\left( {{e^{ - {{\left\langle {\sum\limits_{j = 1}^k {{\lambda _j}} {{\left( - \log \left({{\beta _{P\left(j\right)}^ - } \over q}\right)\right)}\Gamma }} \right\rangle }^{{1 \over \Gamma }}}}}} \right)}},{{\hat s}_{q\left( {{e^{ - {{\left\langle {\sum\limits_{j = 1}^k {{\lambda _j}} {{\left( - \log \left({{\beta _{P\left(j\right)}^ + } \over q}\right)\right)}\Gamma }} \right\rangle }^{{1 \over \Gamma }}}}}} \right)}}} \right]} \cr } } \right).}$$

Without issue, the LIVIFAAOWA operator may have the following characteristics:

**Theorem 7** *(Idempotency property) Let* ${Z_j} = \left([\hat s_{{\theta _j}}^ - ,\hat s_{{\theta _j}}^ + ],[\hat s_{{\beta _j}}^ - ,\hat s_{{\beta _j}}^ + ]\right)$ *and* $j = 1,2, \ldots ,k,$ *be some equal LIVIFNs, i.e.*, ${Z_j} = {Z_1}$, *then* $LIVIFAAOWA\left({Z_1},{Z_2}, \ldots ,{Z_k}\right) = {Z_1}.$

**Theorem 8.** *(Boundedness property) Let* ${Z_j} = \left([\hat s_{{\theta _j}}^ - ,\hat s_{{\theta _j}}^ + ],[\hat s_{{\beta _j}}^ - ,\hat s_{{\beta _j}}^ + ]\right)$. *If* ${Z^ * } = \left([\hat s_{{\theta ^ * }}^ - ,\hat s_{{\theta ^ * }}^ + ],[\hat s_{{\beta ^ * }}^ - ,\hat s_{{\beta ^ * }}^ + ]\right)$ *such that* $\hat s_{{\theta ^ * }}^ - = \mathop {\min }\limits_j \{ \hat s_{{\theta _j}}^ - \} ,\hat s_{{\theta ^ * }}^ + = \mathop {\min }\limits_j \{ \hat s_{{\theta _j}}^ + \}$, $\hat s_{{\beta ^ * }}^ - = \mathop {\max }\limits_j \{ \hat s_{{\beta _j}}^ - \} ,$ $\hat s_{{\beta ^ * }}^ + = \mathop {\max }\limits_j \{ \hat s_{{\beta _j}}^ + \}$ *and* ${Z^{ * * }} = \left([\hat s_{{\theta ^{ * * }}}^ - ,\hat s_{{\theta ^{ * * }}}^ + ],[\hat s_{{\beta ^{ * * }}}^ - ,\hat s_{{\beta ^{ * * }}}^ + ]\right)$ *where* $\hat s_{{\theta ^{ * * }}}^ - = \mathop {\max }\limits_j \{ \hat s_{{\theta _j}}^ - \} ,$ $\hat s_{{\theta ^{ * * }}}^ + = \mathop {\max }\limits_j \{ \hat s_{{\theta _j}}^ + \}$, $\hat s_{{\beta ^{ * * }}}^ - = \mathop {\min }\limits_j \{ \hat s_{{\beta _j}}^ - \} ,$ $\hat s_{{\beta ^{ * * }}}^ + = \mathop {\min }\limits_j \{ \hat s_{{\beta _j}}^ + \} .$ *Then* ${Z^ * } \le LIVIFAAOWA$ $\left({Z_1},{Z_2}, \ldots ,{Z_k}\right) \le$ ${Z^{ * * }}$.

**Theorem 9** *(Monotonicity property) Let* ${Z_j}$ *and* $\acute{Z}_{j}$ *for* $j = 1,2, \ldots ,k,$ *is the LIVIFNs, if for each* $j,$ ${Z_j} \le \acute{Z}_{j}$, *then*

$$LIVIFAAOWA\left({Z_1},{Z_2}, \ldots ,{Z_k}\right) \le LIVIFAAOWA\left(\acute{Z}_{1},\acute{Z}_{2}, \ldots ,\acute{Z}_{k}\right).$$

**Theorem 10** *(Commutativity property) Let* ${Z_j}$ *and* $\acute{Z}_{j}$ *where* $j = 1,2, \ldots ,k$, *be two sets of LIVIFNs, such that for each* $j,$ ${Z_j} \le \acute{Z}_{j}$ *then*

$$LIVIFAAOWA\left({Z_1},{Z_2}, \ldots ,{Z_k}\right) = LIVIFAAOWA\left(\acute{Z}_{1},\acute{Z}_{2}, \ldots ,\acute{Z}_{k}\right).$$

**Definition 10** *If* ${Z_j} = \left([\hat s_{{\theta _j}}^ - ,\hat s_{{\theta _j}}^ + ],[\hat s_{{\beta _j}}^ - ,\hat s_{{\beta _j}}^ + ]\right)$ *where* $j = 1,2, \ldots ,k,$ *is a set of LIVIFNs. Then the LIVIF Aczel-Alsina hybrid average (LIVIFAAHA) operator is a mapping* $LIVIFAAHA:LIVIF{N^k} \to LIVIFN$ *defined as*

$$LIVIFAAHA\left({Z_1},{Z_2}, \ldots ,{Z_m}\right) = \mathop \oplus \limits _{j = 1}^k\left({\lambda _j}Z_{P\left(j\right)}^ \cdot \right).$$

Using Aczel-Alsina operations under LIVIFNs information, the following theorem can be deduced.

**Theorem 11** *If* ${Z_j} = \left([\hat s_{{\theta _j}}^ - ,\hat s_{{\theta _j}}^ + ],[\hat s_{{\beta _j}}^ - ,\hat s_{{\beta _j}}^ + ]\right)$ *where* $j = 1,2, \ldots ,k$ *is a set of LIVIFNs. Accumulated value acquired by LIVIFAAHA operator is again a LIVIFN, i.e.*,

$$\eqalign{&  LIVIFAAHA\left({Z_1},{Z_2}, \ldots ,{Z_k}\right) = \mathop \oplus \limits _{j = 1}^k\left({\lambda _j}Z_{P\left(j\right)}^ \cdot \right)\cr =&  \left( {\matrix{ {\left[ {{{\hat s}_{q\left( {1 - {e^{ - {{\left\langle {\sum\limits_{j = 1}^k {{\lambda _j}} {{\left( - \log \left(1 - {{\theta _{P\left(j\right)}^{\dot - }} \over q}\right)\right)}\Gamma }} \right\rangle }^{{1 \over \Gamma }}}}}} \right)}},{{\hat s}_{q\left( {1 - {e^{ - {{\left\langle {\sum\limits_{j = 1}^k {{\lambda _j}} {{\left( - \log \left(1 - {{\theta _{P\left(j\right)}^{ \cdot + }} \over q}\right)\right)}\Gamma }} \right\rangle }^{{1 \over \Gamma }}}}}} \right)}}} \right],} \cr {\left[ {{{\hat s}_{q\left( {{e^{ - {{\left\langle {\sum\limits_{j = 1}^k {{\lambda _j}} {{\left( - \log \left({{\beta _{P\left(j\right)}^{ \cdot - }} \over q}\right)\right)}\Gamma }} \right\rangle }^{{1 \over \Gamma }}}}}} \right)}},{{\hat s}_{q\left( {{e^{ - {{\left\langle {\sum\limits_{j = 1}^k {{\lambda _j}} {{\left( - \log \left({{\beta _{P\left(j\right)}^{ \cdot + }} \over q}\right)\right)}\Gamma }} \right\rangle }^{{1 \over \Gamma }}}}}} \right)}}} \right]} \cr } } \right)}.$$

**Proof:** This theorem can easily be proved in the same manner as Theorem 2 is.

**Theorem 12** *The LIVIFAAWA and LIVIFAAOWA are special types LIVIVIFAAHA operator*.

**Proof:** (1) Consider $\lambda = {\left({1 \over k},{1 \over k}, \ldots ,{1 \over k}\right)^T}.$ Then

$$\eqalign{LIVIFAAHA\left({Z_1},{Z_2}, \ldots ,{Z_k}\right)=& \, {\lambda _1}Z_{P\left(1\right)}^ \cdot \oplus {\lambda _2}Z_{P\left(2\right)}^ \cdot \oplus \cdots \oplus {\lambda _k}Z_{P\left(k\right)}^ \cdot \cr =& \, {1 \over k}\left(Z_{P\left(1\right)}^ \cdot \oplus Z_{P\left(2\right)}^ \cdot \oplus \cdots \oplus Z_{P\left(k\right)}^ \cdot \right)\cr=& \, {\hat \lambda _1}{Z_1} \oplus {\hat \lambda _2}{Z_2} \oplus \cdots \oplus {\hat \lambda _k}{Z_k}\cr =& \, LIVIFAAWA\left({Z_1},{Z_2}, \ldots ,{Z_k}\right).}$$

(2) Suppose $\hat \lambda = {\left({1 \over k},{1 \over k}, \ldots ,{1 \over k}\right)^T}.$ Then $Z_j^ \cdot = {Z_j}$ where $j = 1,2, \ldots ,k$ and

$$\eqalign{LIVIFAAHA\left({Z_1},{Z_2}, \ldots ,{Z_k}\right)& = {\lambda _1}Z_{P\left(1\right)}^ \cdot \oplus {\lambda _2}Z_{P\left(2\right)}^ \cdot \oplus \cdots \oplus {\lambda _k}Z_{P\left(k\right)}^ \cdot \cr & = {\lambda _1}{Z_{P\left(1\right)}} \oplus {\lambda _2}{Z_{P\left(2\right)}} \oplus \cdots \oplus {\lambda _k}{Z_{P\left(k\right)}}\cr & = LIVIFAAOWA\left({Z_1},{Z_2}, \ldots ,{Z_k}\right).}$$

## Livif aczel-alsina geometric aggregation operators

The following definitions of Aczel-Alsina geometric operators in the context of LIVIFNs are provided in this section:

**Definition 11** *Asume* ${Z_j} = \left([\hat s_{{\theta _j}}^ - ,\hat s_{{\theta _j}}^ + ],[\hat s_{{\beta _j}}^ - ,\hat s_{{\beta _j}}^ + ]\right)$ *Then LIVIFAAWG operator is function* $LIVIFAAWG:LIVIF{N^k} \to LIVIFN$ *defined by:*

$$LIVIFAAWG\left({Z_1},{Z_2}, \ldots ,{Z_k}\right) = \mathop \otimes \limits_{j = 1}^k\left({\lambda _j}{Z_j}\right),$$

*with weight vector is* $\lambda = {\left({\lambda _1},{\lambda _2}, \ldots ,{\lambda _k}\right)^T}$ *also* ${\lambda _j} \in \left[ {0,1} \right]$.

**Theorem 13** *If* ${Z_j} = \left([\hat s_{{\theta _j}}^ - ,\hat s_{{\theta _j}}^ + ],[\hat s_{{\beta _j}}^ - ,\hat s_{{\beta _j}}^ + ]\right)$. *Then LIVIFAAWG operator is again a LIVIFN*,

(3) $$\eqalign{&  LIVIFAAWG\left({Z_1},{Z_2}, \ldots ,{Z_k}\right)\cr =&  \left( {\matrix{ {\left[ {{{\hat s}_{q\left( {{e^{ - {{\left\langle {\sum\limits_{j = 1}^k {{\lambda _j}} {{\left( - \log \left({{\theta _j^ - } \over q}\right)\right)}\Gamma }} \right\rangle }^{{1 \over \Gamma }}}}}} \right)}},{{\hat s}_{q\left( {{e^{ - {{\left\langle {\sum\limits_{j = 1}^k {{\lambda _j}} {{\left( - \log \left({{\theta _j^ + } \over q}\right)\right)}\Gamma }} \right\rangle }^{{1 \over \Gamma }}}}}} \right)}}} \right],} \cr {\left[ {{{\hat s}_{q\left( {1 - {e^{ - {{\left\langle {\sum\limits_{j = 1}^k {{\lambda _j}} {{\left( - \log \left(1 - {{\beta _j^ - } \over q}\right)\right)}\Gamma }} \right\rangle }^{{1 \over \Gamma }}}}}} \right)}},{{\hat s}_{q\left( {1 - {e^{ - {{\left\langle {\sum\limits_{j = 1}^k {{\lambda _j}} {{\left( - \log \left(1 - {{\beta _j^ + } \over q}\right)\right)}\Gamma }} \right\rangle }^{{1 \over \Gamma }}}}}} \right)}}} \right]} \cr } } \right)}$$

*where weight vector is* $\lambda = {\left({\lambda _1},{\lambda _2}, \ldots ,{\lambda _k}\right)^T}$.

**Proof:** We employ mathematical induction to demonstrate this theorem.

(i) If we assume that $k = 2,$ we may calculate that using the Aczel-Alsina operations of LIVIFNs.

$${{\lambda _1}{Z_1} =
\left({\matrix{ {\left[ {{{\hat s}_{q\left( {{e^{ - {{\left\langle {{\lambda _1}{{\left( - \log \left({{\theta _1^ - } \over q}\right)\right)}\Gamma }} \right\rangle }^{{1 \over \Gamma }}}}}} \right)}},{{\hat s}_{q\left( {{e^{ - {{\left\langle {{\lambda _1}{{\left( - \log \left({{\theta _1^ + } \over q}\right)\right)}\Gamma }} \right\rangle }^{{1 \over \Gamma }}}}}} \right)}}} \right],} \cr  {\left[ {{{\hat s}_{q\left( {1 - {e^{ - {{\left\langle {{\lambda _1}{{\left( - \log \left(1 - {{\beta _1^ - } \over q}\right)\right)}\Gamma }} \right\rangle }^{{1 \over \Gamma }}}}}} \right)}},{{\hat s}_{q\left( {1 - {e^{ - {{\left\langle {{\lambda _1}{{\left( - \log \left(1 - {{\beta _1^ + } \over q}\right)\right)}\Gamma }} \right\rangle }^{{1 \over \Gamma }}}}}} \right)}}} \right]}} }\right)}$$

$${{\lambda _2}{Z_2} = \left( {\matrix{ {\left[ {{{\hat s}_{q\left( {{e^{ - {{\left\langle {{\lambda _2}{{\left( - \log \left({{\theta _2^ - } \over q}\right)\right)}\Gamma }} \right\rangle }^{{1 \over \Gamma }}}}}} \right)}},{{\hat s}_{q\left( {{e^{ - {{\left\langle {{\lambda _2}{{\left( - \log \left({{\theta _2^ + } \over q}\right)\right)}\Gamma }} \right\rangle }^{{1 \over \Gamma }}}}}} \right)}}} \right],} \cr  {\left[ {{{\hat s}_{q\left( {1 - {e^{ - {{\left\langle {{\lambda _2}{{\left( - \log \left(1 - {{\beta _2^ - } \over q}\right)\right)}\Gamma }} \right\rangle }^{{1 \over \Gamma }}}}}} \right)}},{{\hat s}_{q\left( {1 - {e^{ - {{\left\langle {{\lambda _2}{{\left( - \log \left(1 - {{\beta _2^ + } \over q}\right)\right)}\Gamma }} \right\rangle }^{{1 \over \Gamma }}}}}} \right)}}} \right]} \cr  } } \right).}$$

Applying definition 7, we obtain

$$\eqalign{&  LIVIFAAWG\left({Z_1},{Z_2}\right) = {\lambda _1}{Z_1} \otimes {\lambda _2}{Z_2}\cr =&  \left( {\matrix{ {\left[ {{{\hat s}_{q\left( {{e^{ - {{\left\langle {{\lambda _1}{{\left( - \log \left({{\theta _1^ - } \over q}\right)\right)}\Gamma }} \right\rangle }^{{1 \over \Gamma }}}}}} \right)}},{{\hat s}_{q\left( {{e^{ - {{\left\langle {{\lambda _1}{{\left( - \log \left({{\theta _1^ + } \over q}\right)\right)}\Gamma }} \right\rangle }^{{1 \over \Gamma }}}}}} \right)}}} \right],} \cr {\left[ {{{\hat s}_{q\left( {1 - {e^{ - {{\left\langle {{\lambda _1}{{\left( - \log \left(1 - {{\beta _1^ - } \over q}\right)\right)}\Gamma }} \right\rangle }^{{1 \over \Gamma }}}}}} \right)}},{{\hat s}_{q\left( {1 - {e^{ - {{\left\langle {{\lambda _1}{{\left( - \log \left(1 - {{\beta _1^ + } \over q}\right)\right)}\Gamma }} \right\rangle }^{{1 \over \Gamma }}}}}} \right)}}} \right]} \cr } } \right)}$$

$$\eqalign{  &  \otimes \left( \matrix{  \;\;\;\;\;\;\left[ {{{\hat s}_{q\left( {{e^{ - {{\left\langle {{\lambda _2}\left( { - \log \left( {{{\theta _2^ - } \over q}} \right)} \right)\Gamma } \right\rangle }^{{1 \over \Gamma }}}}}} \right)}},{{\hat s}_{q\left( {{e^{ - {{\left\langle {{\lambda _2}\left( { - \log \left( {{{\theta _2^ + } \over q}} \right)} \right)\Gamma } \right\rangle }^{{1 \over \Gamma }}}}}} \right)}}} \right], \cr   \left[ {{{\hat s}_{q\left( {1 - {e^{ - {{\left\langle {{\lambda _2}\left( { - \log \left( {1 - {{\beta _2^ - } \over q}} \right)} \right)\Gamma } \right\rangle }^{{1 \over \Gamma }}}}}} \right)}},{{\hat s}_{q\left( {1 - {e^{ - {{\left\langle {{\lambda _2}\left( { - \log \left( {1 - {{\beta _2^ + } \over q}} \right)} \right)\Gamma } \right\rangle }^{{1 \over \Gamma }}}}}} \right)}}} \right] \cr}  \right)  \cr   &  = \left( \matrix{ \;\;\;\;\;\; \left[ {{{\hat s}_{q\left( {{e^{ - {{\left\langle {{\lambda _1}\left( { - \log \left( {{{\theta _1^ - } \over q}} \right)} \right)\Gamma  + {\lambda _2}\left( { - \log \left( {{{\theta _2^ - } \over q}} \right)} \right)\Gamma } \right\rangle }^{{1 \over \Gamma }}}}}} \right)}},{{\hat s}_{q\left( {{e^{ - {{\left\langle {{\lambda _1}\left( { - \log \left( {{{\theta _1^ + } \over q}} \right)} \right)\Gamma  + {\lambda _2}\left( { - \log \left( {{{\theta _2^ + } \over q}} \right)} \right)\Gamma } \right\rangle }^{{1 \over \Gamma }}}}}} \right)}}} \right], \cr   \left[ {{{\hat s}_{q\left( {1 - {e^{ - {{\left\langle {{\lambda _1}\left( { - \log \left( {1 - {{\beta _1^ - } \over q}} \right)} \right)\Gamma  + {\lambda _2}\left( { - \log \left( {1 - {{\beta _2^ - } \over q}} \right)} \right)\Gamma } \right\rangle }^{{1 \over \Gamma }}}}}} \right)}},{{\hat s}_{q\left( {1 - {e^{ - {{\left\langle {{\lambda _1}\left( { - \log \left( {1 - {{\beta _1^ + } \over q}} \right)} \right)\Gamma  + {\lambda _2}\left( { - \log \left( {1 - {{\beta _2^ + } \over q}} \right)} \right)\Gamma } \right\rangle }^{{1 \over \Gamma }}}}}} \right)}}} \right] \cr}  \right)  \cr   &  = \left( \matrix{ \;\;\; \left[ {{{\hat s}_{q\left( {{e^{ - {{\left\langle {\sum\limits_{j = 1}^2 {{\lambda _j}} \left( { - \log \left( {{{\theta _j^ - } \over q}} \right)} \right)\Gamma } \right\rangle }^{{1 \over \Gamma }}}}}} \right)}},{{\hat s}_{q\left( {{e^{ - {{\left\langle {\sum\limits_{j = 1}^2 {{\lambda _j}} \left( { - \log \left( {{{\theta _j^ + } \over q}} \right)} \right)\Gamma } \right\rangle }^{{1 \over \Gamma }}}}}} \right)}}} \right], \cr   \left[ {{{\hat s}_{q\left( {1 - {e^{ - {{\left\langle {\sum\limits_{j = 1}^2 {{\lambda _j}} \left( { - \log \left( {1 - {{\beta _j^ - } \over q}} \right)} \right)\Gamma } \right\rangle }^{{1 \over \Gamma }}}}}} \right)}},{{\hat s}_{q\left( {1 - {e^{ - {{\left\langle {\sum\limits_{j = 1}^2 {{\lambda _j}} \left( { - \log \left( {1 - {{\beta _j^ + } \over q}} \right)} \right)\Gamma } \right\rangle }^{{1 \over \Gamma }}}}}} \right)}}} \right] \cr}  \right). \cr} $$

So, for $k = 2$, Eq. (3) is accurate.

(ii) If Eq. (3) holds, when $k = m$, therefore

$$\eqalign{& LIVIFAAWG\left({Z_1},{Z_2}, \ldots ,{Z_m}\right) = \otimes _{j = 1}^m\left({\lambda _j}{Z_j}\right)\cr =&  \left( {\matrix{ {\left[ {{{\hat s}_{q\left( {{e^{ - {{\left\langle {\sum\limits_{j = 1}^m {{\lambda _j}} {{\left( - \log \left({{\theta _j^ - } \over q}\right)\right)}\Gamma }} \right\rangle }^{{1 \over \Gamma }}}}}} \right)}},{{\hat s}_{q\left( {{e^{ - {{\left\langle {\sum\limits_{j = 1}^m {{\lambda _j}} {{\left( - \log \left({{\theta _j^ + } \over q}\right)\right)}\Gamma }} \right\rangle }^{{1 \over \Gamma }}}}}} \right)}}} \right],} \cr {\left[ {{{\hat s}_{q\left( {1 - {e^{ - {{\left\langle {\sum\limits_{j = 1}^m {{\lambda _j}} {{\left( - \log \left(1 - {{\beta _j^ - } \over q}\right)\right)}\Gamma }} \right\rangle }^{{1 \over \Gamma }}}}}} \right)}},{{\hat s}_{q\left( {1 - {e^{ - {{\left\langle {\sum\limits_{j = 1}^m {{\lambda _j}} {{\left( - \log \left(1 - {{\beta _j^ + } \over q}\right)\right)}\Gamma }} \right\rangle }^{{1 \over \Gamma }}}}}} \right)}}} \right]}} } \right)}.$$

(iii) Let us take $k = m + 1$, then

$$\eqalign{&  LIVIFAAWG\left({Z_1},{Z_2}, \ldots ,{Z_{m + 1}}\right) = \otimes _{j = 1}^m\left({\lambda _j}{Z_j}\right) \otimes {\lambda _{m + 1}}{Z_{m + 1}}\cr =&  \left( {\matrix{ {\left[ {{{\hat s}_{q\left( {{e^{ - {{\left\langle {\sum\limits_{j = 1}^m {{\lambda _j}} {{\left( - \log \left({{\theta _j^ - } \over q}\right)\right)}\Gamma }} \right\rangle }^{{1 \over \Gamma }}}}}} \right)}},{{\hat s}_{q\left( {{e^{ - {{\left\langle {\sum\limits_{j = 1}^m {{\lambda _j}} {{\left( - \log \left({{\theta _j^ + } \over q}\right)\right)}\Gamma }} \right\rangle }^{{1 \over \Gamma }}}}}} \right)}}} \right],} \cr {\left[ {{{\hat s}_{q\left( {1 - {e^{ - {{\left\langle {\sum\limits_{j = 1}^m {{\lambda _j}} {{\left( - \log \left(1 - {{\beta _j^ - } \over q}\right)\right)}\Gamma }} \right\rangle }^{{1 \over \Gamma }}}}}} \right)}},{{\hat s}_{q\left( {1 - {e^{ - {{\left\langle {\sum\limits_{j = 1}^m {{\lambda _j}} {{\left( - \log \left(1 - {{\beta _j^ + } \over q}\right)\right)}\Gamma }} \right\rangle }^{{1 \over \Gamma }}}}}} \right)}}} \right]} \cr } } \right)}$$

$$\eqalign{&  \otimes \left( {\matrix{ {\left[ {{{\hat s}_{q\left( {{e^{ - {{\left\langle {{\lambda _{m + 1}}{{\left( - \log \left({{\theta _{m + 1}^ - } \over q}\right)\right)}\Gamma }} \right\rangle }^{{1 \over \Gamma }}}}}} \right)}},{{\hat s}_{q\left( {{e^{ - {{\left\langle {{\lambda _{m + 1}}{{\left( - \log \left({{\theta _{m + 1}^ + } \over q}\right)\right)}\Gamma }} \right\rangle }^{{1 \over \Gamma }}}}}} \right)}}} \right],} \cr {\left[ {{{\hat s}_{q\left( {1 - {e^{ - {{\left\langle {{\lambda _{m + 1}}{{\left( - \log \left(1 - {{\beta _{m + 1}^ - } \over q}\right)\right)}\Gamma }} \right\rangle }^{{1 \over \Gamma }}}}}} \right)}},{{\hat s}_{q\left( {1 - {e^{ - {{\left\langle {{\lambda _{m + 1}}{{\left( - \log \left(1 - {{\beta _{m + 1}^ + } \over q}\right)\right)}\Gamma }} \right\rangle }^{{1 \over \Gamma }}}}}} \right)}}} \right]} \cr } } \right)\cr&  = \left( {\matrix{ {\left[ {{{\hat s}_{q\left( {{e^{ - {{\left\langle {\sum\limits_{j = 1}^{m + 1} {{\lambda _j}} {{\left( - \log \left({{\theta _j^ - } \over q}\right)\right)}\Gamma }} \right\rangle }^{{1 \over \Gamma }}}}}} \right)}},{{\hat s}_{q\left( {{e^{ - {{\left\langle {\sum\limits_{j = 1}^{m + 1} {{\lambda _j}} {{\left( - \log \left({{\theta _j^ + } \over q}\right)\right)}\Gamma }} \right\rangle }^{{1 \over \Gamma }}}}}} \right)}}} \right],} \cr {\left[ {{{\hat s}_{q\left( {1 - {e^{ - {{\left\langle {\sum\limits_{j = 1}^{m + 1} {{\lambda _j}} {{\left( - \log \left(1 - {{\beta _j^ - } \over q}\right)\right)}\Gamma }} \right\rangle }^{{1 \over \Gamma }}}}}} \right)}},{{\hat s}_{q\left( {1 - {e^{ - {{\left\langle {\sum\limits_{j = 1}^{m + 1} {{\lambda _j}} {{\left( - \log \left(1 - {{\beta _j^ + } \over q}\right)\right)}\Gamma }} \right\rangle }^{{1 \over \Gamma }}}}}} \right)}}} \right]} \cr } } \right).}$$

Thus, Eq. (3) is true for any positive integer k.

The LIVIFAAWG operator may have the following properties without any issue.

**Theorem 14** *(Idempotency property) Let* ${Z_j} = \left([\hat s_{{\theta _j}}^ - ,\hat s_{{\theta _j}}^ + ],[\hat s_{{\beta _j}}^ - ,\hat s_{{\beta _j}}^ + ]\right)$, *then*

$$LIVIFAAWG\left({Z_1},{Z_2}, \ldots ,{Z_k}\right) = {Z_1}.$$

**Theorem 15** *(Boundedness property) Let* ${Z_j} = \left([\hat s_{{\theta _j}}^ - ,\hat s_{{\theta _j}}^ + ],[\hat s_{{\beta _j}}^ - ,\hat s_{{\beta _j}}^ + ]\right)$ *where* $j = 1,2, \ldots ,k,$ *is LIVIFNs. If* ${Z^ * } = \left([\hat s_{{\theta ^ * }}^ - ,\hat s_{{\theta ^ * }}^ + ],[\hat s_{{\beta ^ * }}^ - ,\hat s_{{\beta ^ * }}^ + ]\right)$ *such that* $\hat s_{{\theta ^ * }}^ - = \mathop {\min }\limits_j \{ \hat s_{{\theta _j}}^ - \} ,\hat s_{{\theta ^ * }}^ + = \mathop {\min }\limits_j \{ \hat s_{{\theta _j}}^ + \}$, $\hat s_{{\beta ^ * }}^ - = \mathop {\max }\limits_j \{ \hat s_{{\beta _j}}^ - \} ,$ $\hat s_{{\beta ^ * }}^ + = \mathop {\max }\limits_j \{ \hat s_{{\beta _j}}^ + \}$ *and* ${Z^{ * * }} = \left([\hat s_{{\theta ^{ * * }}}^ - ,\hat s_{{\theta ^{ * * }}}^ + ],[\hat s_{{\beta ^{ * * }}}^ - ,\hat s_{{\beta ^{ * * }}}^ + ]\right)$ *where* $\hat s_{{\theta ^{ * * }}}^ - = \mathop {\max }\limits_j \{ \hat s_{{\theta _j}}^ - \} ,$ $\hat s_{{\theta ^{ * * }}}^ + = \mathop {\max }\limits_j \{ \hat s_{{\theta _j}}^ + \}$, $\hat s_{{\beta ^{ * * }}}^ - = \mathop {\min }\limits_j \{ \hat s_{{\beta _j}}^ - \} ,$ $\hat s_{{\beta ^{ * * }}}^ + = \mathop {\min }\limits_j \{ \hat s_{{\beta _j}}^ + \} .$*Then* ${Z^ * } \le LIVIFAAWG\left({Z_1},{Z_2}, \ldots ,{Z_k}\right) \le$ ${Z^{ * * }}$.

**Theorem 16** *(Monotonicity property) Let* ${Z_j}$ *and* $\acute{Z_j}$ *where* $j = 1,2, \ldots ,k,$ *is LIVIFNs, if for each* $j = 1,2, \ldots ,k,$ $\acute{Z}_{j} \le Z_j$, *then*

$$LIVIFAAWG\left({Z_1},{Z_2}, \ldots ,{Z_k}\right) \le LIVIFAAWG\left(\acute{Z}_{1},\acute{Z}_{2}, \ldots ,\acute{Z}_{k}\right).$$

Next, we present the LIVIF Aczel-Alsina ordered weighted Geometric (LIVIFAAOWG) operator.

**Definition 12** *Let* ${Z_j} = \left([\hat s_{{\theta _j}}^ - ,\hat s_{{\theta _j}}^ + ],[\hat s_{{\beta _j}}^ - ,\hat s_{{\beta _j}}^ + ]\right)$ *where* $j = 1,2, \ldots ,k,$ *is LIVIFNs. Then LIVIF Aczel-Alsina ordered weighted geometric (LIVIFAAOWG) operator is a mapping* $LIVIFAAOWG:LIVIF{N^k} \to LIVIFN$ *with relating vector* $\lambda = {\left({\lambda _1},{\lambda _2}, \ldots ,{\lambda _k}\right)^T},$ *and* $\sum\nolimits_{j = 1}^k {{\lambda _j}} = 1.$ *Therefore*, $LIVIFAAOWG\left({Z_1},{Z_2}, \ldots ,{Z_k}\right) = \otimes _{j = 1}^k\left({\lambda _j}{Z_{P\left(j\right)}}\right).$

**Theorem 17** *Assume* ${Z_j} = \left([\hat s_{{\theta _j}}^ - ,\hat s_{{\theta _j}}^ + ],[\hat s_{{\beta _j}}^ - ,\hat s_{{\beta _j}}^ + ]\right)$ *where* $j = 1,2, \ldots ,k,$ *is LIVIFNs. Then LIVIFAAOWG is a function* $LIVIFAAOWG:LIVIF{N^k} \to LIVIFN$ *and* $\sum\nolimits_{j = 1}^k {{\lambda _j}} = 1.$ *Therefore*,

$$\eqalign{ &  LIVIFAAOWG\left({Z_1},{Z_2}, \ldots ,{Z_k}\right) = \otimes _{j = 1}^k\left({\lambda _j}{Z_{P\left(j\right)}}\right)\cr = &  \left( {\matrix{ {\left[ {{{\hat s}_{q\left( {{e^{ - {{\left\langle {\sum\limits_{j = 1}^k {{\lambda _j}} {{\left( - \log \left({{\theta _{P\left(j\right)}^ - } \over q}\right)\right)}\Gamma }} \right\rangle }^{{1 \over \Gamma }}}}}} \right)}},{{\hat s}_{q\left( {{e^{ - {{\left\langle {\sum\limits_{j = 1}^k {{\lambda _j}} {{\left( - \log \left({{\theta _{P\left(j\right)}^ + } \over q}\right)\right)}\Gamma }} \right\rangle }^{{1 \over \Gamma }}}}}} \right)}}} \right],} \cr {\left[ {{{\hat s}_{q\left( {1 - {e^{ - {{\left\langle {\sum\limits_{j = 1}^k {{\lambda _j}} {{\left( - \log \left(1 - {{\beta _{P\left(j\right)}^ - } \over q}\right)\right)}\Gamma }} \right\rangle }^{{1 \over \Gamma }}}}}} \right)}},{{\hat s}_{q\left( {1 - {e^{ - {{\left\langle {\sum\limits_{j = 1}^k {{\lambda _j}} {{\left( - \log \left(1 - {{\beta _{P\left(j\right)}^ + } \over q}\right)\right)}\Gamma }} \right\rangle }^{{1 \over \Gamma }}}}}} \right)}}} \right]}} } \right).}$$

LIVIFAAOWG operator may have the following properties without any issue.

**Theorem 18** *(Idempotency property) Let* ${Z_j} = \left([\hat s_{{\theta _j}}^ - ,\hat s_{{\theta _j}}^ + ],[\hat s_{{\beta _j}}^ - ,\hat s_{{\beta _j}}^ + ]\right)$ $\left(j = 1,2, \ldots ,k\right)$ *be LIVIFNs, i.e., for each* $j,$ ${Z_j} = {Z_1}$, *then* $LIVIFAAOWG\left({Z_1},{Z_2}, \ldots ,{Z_k}\right) = {Z_1}.$

**Theorem 19** *(Boundedness property) Let* ${Z_j} = \left([\hat s_{{\theta _j}}^ - ,\hat s_{{\theta _j}}^ + ],[\hat s_{{\beta _j}}^ - ,\hat s_{{\beta _j}}^ + ]\right)$ *where* $j = 1,2, \ldots ,k,$ *be a collection of LIVIFNs. If* ${Z^ * } = \left([\hat s_{{\theta ^ * }}^ - ,\hat s_{{\theta ^ * }}^ + ],[\hat s_{{\beta ^ * }}^ - ,\hat s_{{\beta ^ * }}^ + ]\right)$ *such that* $\hat s_{{\theta ^ * }}^ - = \mathop {\min }\limits_j \{ \hat s_{{\theta _j}}^ - \} ,\hat s_{{\theta ^ * }}^ + = \mathop {\min }\limits_j \{ \hat s_{{\theta _j}}^ + \}$, $\hat s_{{\beta ^ * }}^ - = \mathop {\max }\limits_j \{ \hat s_{{\beta _j}}^ - \} ,$ $\hat s_{{\beta ^ * }}^ + = \mathop {\max }\limits_j \{ \hat s_{{\beta _j}}^ + \}$ *and* ${Z^{ * * }} = \left([\hat s_{{\theta ^{ * * }}}^ - ,\hat s_{{\theta ^{ * * }}}^ + ],[\hat s_{{\beta ^{ * * }}}^ - ,\hat s_{{\beta ^{ * * }}}^ + ]\right)$ *where* $\hat s_{{\theta ^{ * * }}}^ - = \mathop {\max }\limits_j \{ \hat s_{{\theta _j}}^ - \} ,$ $\hat s_{{\theta ^{ * * }}}^ + = \mathop {\max }\limits_j \{ \hat s_{{\theta _j}}^ + \}$, $\hat s_{{\beta ^{ * * }}}^ - = \mathop {\min }\limits_j \{ \hat s_{{\beta _j}}^ - \} ,$ $\hat s_{{\beta ^{ * * }}}^ + = \mathop {\min }\limits_j \{ \hat s_{{\beta _j}}^ + \} .$ *Then* ${Z^ * } \le LIVIFAAOWG$ $\left({Z_1},{Z_2}, \ldots ,{Z_k}\right) \le$ ${Z^{ * * }}.$

**Theorem 20** *(Monotonicity property) If* ${Z_j}$ *and* $\acute{Z}_j$ *where* $j = 1, \ldots ,k,$ *are LIVIFNs and* ${Z_j} \le \acute{Z}_j$ *for all* $j$, *then*

$LIVIFAAOWG\left({Z_1},{Z_2}, \ldots ,{Z_k}\right) \le LIVIFAAOWG\left(\acute{Z}_1,\acute{Z}_2, \ldots ,\acute{Z}_k\right).$

**Theorem 21** *(Commutativity property) Let* ${Z_j}$ *and* $\acute{Z}_j$ *such that* ${Z_j} \le \acute{Z}_j$ *for all* $j$, *then*

$LIVIFAAOWG\left({Z_1},{Z_2}, \ldots ,{Z_k}\right) = LIVIFAAOWG\left(\acute{Z}_1,\acute{Z}_2, \ldots ,\acute{Z}_k\right).$

**Definition 13** *Let* ${Z_j} = \left([\hat s_{{\theta _j}}^ - ,\hat s_{{\theta _j}}^ + ],[\hat s_{{\beta _j}}^ - ,\hat s_{{\beta _j}}^ + ]\right)$ *where* $j = 1,2, \ldots ,k,$ *be a collection of LIVIFNs. Then LIVIFAAHG fuction* $LIVIFAAHG:LIVIF{N^k} \to LIVIFN$ *defined by*

$$LIVIFAAHG\left({Z_1},{Z_2}, \ldots ,{Z_k}\right) = \otimes _{j = 1}^k\left({\lambda _j}Z_{P\left(j\right)}^ \cdot \right).$$

The following theorem may be derived utilising Aczel-Alsina operations under LIVIFNs information.

**Theorem 22** *Assume* ${Z_j}$ $\left(j = 1,2, \ldots ,k\right)$ *is LIVIFNs. Accumulated obtained by LIVIFAAHG is again a LIVIFN, and*

$$\eqalign{& LIVIFAAHG\left({Z_1},{Z_2}, \ldots ,{Z_k}\right) = \otimes _{j = 1}^k\left({\lambda _j}Z_{P\left(j\right)}^ \cdot \right)\cr =& \left( {\matrix{ {\left[ {{{\hat s}_{q\left( {1 - {e^{ - {{\left\langle {\sum\limits_{j = 1}^k {{\lambda _j}} {{\left( - \log \left({{\theta _{P\left(j\right)}^{ \cdot - }} \over q}\right)\right)}\Gamma }} \right\rangle }^{{1 \over \Gamma }}}}}} \right)}},{{\hat s}_{q\left( {1 - {e^{ - {{\left\langle {\sum\limits_{j = 1}^k {{\lambda _j}} {{\left( - \log \left({{\theta _{P\left(j\right)}^{ \cdot + }} \over q}\right)\right)}\Gamma }} \right\rangle }^{{1 \over \Gamma }}}}}} \right)}}} \right],} \cr {\left[ {{{\hat s}_{q\left( {{e^{ - {{\left\langle {\sum\limits_{j = 1}^k {{\lambda _j}} {{\left( - \log \left(1 - {{\beta _{P\left(j\right)}^{ \cdot - }} \over q}\right)\right)}\Gamma }} \right\rangle }^{{1 \over \Gamma }}}}}} \right)}},{{\hat s}_{q\left( {{e^{ - {{\left\langle {\sum\limits_{j = 1}^k {{\lambda _j}} {{\left( - \log \left(1 - {{\beta _{P\left(j\right)}^{ \cdot + }} \over q}\right)\right)}\Gamma }} \right\rangle }^{{1 \over \Gamma }}}}}} \right)}}} \right]}} } \right).}$$

**Proof:** Just like Theorem 12, we can easily prove this theorem.

**Theorem 23** *The LIVIFAAHG operator has special situations known as LIVIFAAWG operator and LIVIFAAOWG operator*.

**Proof:** (1) Suppose that $\lambda = {\left({1 \over k},{1 \over k}, \ldots ,{1 \over k}\right)^T}.$ Then

$$\eqalign{ LIVIFAAHG\left({Z_1},{Z_2}, \ldots ,{Z_k}\right) & = {\lambda _1}Z_{P\left(1\right)}^ \cdot \otimes {\lambda _2}Z_{P\left(2\right)}^ \cdot \otimes \cdots \mathop \otimes \limits_m \lambda Z_{P\left(k\right)}^ \cdot \cr & = {1 \over k}\big(Z_{P\left(1\right)}^ \cdot \otimes Z_{P\left(2\right)}^ \cdot \otimes \cdots \otimes Z_{P\left(k\right)}^ \cdot \cr &  = {\hat \lambda _1}{Z_1} \otimes {\hat \lambda _2}{Z_2} \otimes \cdots \otimes {\hat \lambda _k}{Z_k} \cr &  = LIVIFAAWG\left({Z_1},{Z_2}, \ldots ,{Z_k}\right).}$$

(2) Let $\hat \lambda$ = $({1 \over k},{1 \over k}, \ldots ,{1 \over k}{)^T}.$ Then $Z_j^ \cdot = {Z_j}$ $\left(j = 1,2, \ldots ,k\right)$ and

$$\eqalign{LIVIFAAHG\left({Z_1},{Z_2}, \ldots ,{Z_k}\right) & = {\lambda _1}Z_{P\left(1\right)}^ \cdot \otimes {\lambda _2}Z_{P\left(2\right)}^ \cdot \otimes \cdots \otimes {\lambda _k}Z_{P\left(k\right)}^ \cdot \cr & = {\lambda _1}{Z_{P\left(1\right)}} \otimes {\lambda _2}{Z_{P\left(2\right)}} \otimes \cdots \otimes {\lambda _k}{Z_{P\left(k\right)}}\cr & = LIVIFAAOWG\left({Z_1},{Z_2}, \ldots ,{Z_k}\right).}$$

## Madm approach based with livif information

Therefore in part, we will use a MADM issue in a LIVIF environment to apply the suggested operators. To give decision choice, MADM techniques analyse tradeoffs of alternative exhibits across many aspects. Attribute values or performance metrics are the essential data for the MADM approach. A MADM issue is a task that entails selecting a workable compromise choice from among all feasible possibilities based on numerous qualities.

In this instance, we may propose a MADM approach that manages LIVIF Aczel-Alsina aggregation operations. Assume that $\acute{E} = \{ \acute{E}_{1},\acute{E}_{2}, \ldots ,\acute{E}_{m}\}$ are the discretely arranged alternatives, the discretely arranged attributes are $\hat C = \{ {\hat c_1},{\hat c_2}, \ldots ,{\hat c_n}\}$. ${\hat c_j}(j = 1,2, \ldots ,n\}$ is $\lambda = \{ {\lambda _1},{\lambda _2}, \ldots ,{\lambda _n}\}$ such that ${\lambda _j} \in [0,1]$ and $\sum\nolimits_{j = 1}^n {{\lambda _j}} = 1$. Assume ${T_q} = {\left({Z_{ij}}\right)_{m \times n}} = {\left([\hat s_{{\theta _{ij}}}^ - ,\hat s_{{\theta _{ij}}}^ + ],[\hat s_{{\beta _{ij}}}^ - ,\hat s_{{\beta _{ij}}}^ + ]\right)_{m \times n}}$ denote the LIVIF decision matrix (Table 1), where $[\hat s_{{\theta _{ij}}}^ - ,\hat s_{{\theta _{ij}}}^ + ]$ denotes MD with alternatives $\acute{E}_{i}$ also with attributes ${\hat c_j}$, and $[\hat s_{{\beta _{ij}}}^ - ,\hat s_{{\beta _{ij}}}^ + ]$ denotes NMD with alternative $\acute{E}_{i}$ and attribute ${\hat c_j}$, and $0 \le \hat s_{{\theta _{ij}}}^ + + \hat s_{{\beta _{ij}}}^ + \le q,\left(i = 1,2, \ldots ,m\right)$ and $\left(j = 1,2, \ldots ,n\right)$.

**Table 1:** LIVIF decision matrix.

		${\hat c_1}$	${\hat c_2}$	$\cdots$	${\hat c_n}$
	$\acute{E}_{1}$	${Z_{11}}$	${Z_{12}}$	$\cdots$	${Z_{1n}}$
${T_q} = {\left({Z_{ij}}\right)_{m \times n}} =$	$\acute{E}_{2}$	${Z_{21}}$	${Z_{22}}$	$\cdots$	${Z_{2n}}$
	$\vdots$	$\vdots$	$\vdots$	$\ddots$	$\vdots$
	$\acute{E}_{m}$	${Z_{m1}}$	${Z_{m2}}$	$\cdots$	${Z_{mn}}$

### MADM model for LIVIF information based on proposed operators

Using the LIVIFAAWA and LIVIFAAWG operators, we construct an approach for determining the optimal alternative(s) for the MADM problem, which consist of different steps below:

**Step 1.** Considering MADM LIVIFNs, first, develop the LIVIF decision table ${T_q} = {\left({Z_{ij}}\right)_{m \times n}}$, and each entry $\acute{E}_{m}$ regarding the attribute ${\hat c_n}$.

**Step 2.** We make use of the following equation to convert the LIVIF decision matrix ${T_q} = {\left({Z_{ij}}\right)_{m \times n}}$ into the normalised one $\widetilde{T_q} = {\left(\acute{E_{ij}}\right)_{m \times n}}$,

(4) $$\acute{E_{ij}} = \left\{ {\matrix{ {{Z_{ij}},j \in B} \cr {Z_{ij}^c,j \in C} \cr } } \right.$$

where $Z_{ij}^c$ denote the complement of ${Z_{ij}}.$

**Step 3.** Utilize the LIVIFAAWA/LIVIFAAWG operator to aggregate the individual linguistic interval valued intuitionistic fuzzy decision matrices presented by the $k$ DMs.

$$\eqalign{{\widetilde{\acute {E}}_{ij}} =\,&  LIVIFAAWA\left(\acute {E}_{ij}^{\left(1\right)},\acute{E}_{ij}^{\left(2\right)}, \ldots ,\acute{E}_{ij}^{\left(l\right)}\right)\cr =&  \left( {\left[ {{{\hat s}_{q\left( {1 - {e^{ - {{\left\langle {\sum\limits_{j = 1}^l {{{\bar {\lambda} }_j}} {{\left( - \log \left(1 - {{{{\left(\theta _{ij}^ - \right)}^{\left(k\right)}}} \over q}\right)\right)}\Gamma }} \right\rangle }^{{1 \over \Gamma }}}}}} \right)}},{{\hat s}_{q\left( {1 - {e^{ - {{\left\langle {\sum\limits_{j = 1}^l {{{\bar \lambda }_{ij}}} {{\left( - \log \left(1 - {{{{\left(\theta _{ij}^ + \right)}^{\left(k\right)}}} \over q}\right)\right)}\Gamma }} \right\rangle }^{{1 \over \Gamma }}}}}} \right)}}} \right]} \right.,\cr &  \left. {\left[ {{{\hat s}_{q\left( {{e^{ - {{\left\langle {\sum\limits_{j = 1}^l {{{\bar \lambda }_{ij}}} {{\left( - \log \left({{{{\left(\beta _{ij}^ - \right)}^{\left(k\right)}}} \over q}\right)\right)}\Gamma }} \right\rangle }^{{1 \over \Gamma }}}}}} \right)}},{{\hat s}_{q\left( {{e^{ - {{\left\langle {\sum\limits_{j = 1}^l {{{\bar \lambda }_{ij}}} {{\left( - \log \left({{{{\left(\beta _{ij}^ + \right)}^{\left(k\right)}}} \over q}\right)\right)}\Gamma }} \right\rangle }^{{1 \over \Gamma }}}}}} \right)}}} \right]} \right).}$$

or

$${\eqalign{{\widetilde {\acute{E}}_{ij}} =\,&  LIVIFAAWG\left(\acute {E}_{ij}^{\left(1\right)},\acute {E}_{ij}^{\left(2\right)}, \ldots ,\acute {E}_{ij}^l\right)\cr =&  \left( {\left[ {{{\hat s}_{q\left( {{e^{ - {{\left\langle {\sum\limits_{j = 1}^l {{\lambda _j}} {{\left( - \log \left({{{{\left(\theta _{ij}^ - \right)}^{\left(k\right)}}} \over q}\right)\right)}\Gamma }} \right\rangle }^{{1 \over \Gamma }}}}}} \right)}},{{\hat s}_{q\left( {{e^{ - {{\left\langle {\sum\limits_{j = 1}^l {{\lambda _{ij}}} {{\left( - \log \left({{{{\left(\theta _{ij}^ + \right)}^{\left(k\right)}}} \over q}\right)\right)}\Gamma }} \right\rangle }^{{1 \over \Gamma }}}}}} \right)}}} \right]} \right.,\cr & \left. {\left[ {{{\hat s}_{q\left( {1 - {e^{ - {{\left\langle {\sum\limits_{j = 1}^l {{\lambda _j}} {{\left( - \log \left(1 - {{{{\left(\beta _{ij}^ - \right)}^{\left(k\right)}}} \over q}\right)\right)}\Gamma }} \right\rangle }^{{1 \over \Gamma }}}}}} \right)}},{{\hat s}_{q\left( {1 - {e^{ - {{\left\langle {\sum\limits_{j = 1}^l {{\lambda _j}} {{\left( - \log \left(1 - {{{{\left(\beta _{ij}^ + \right)}^{\left(k\right)}}} \over q}\right)\right)}\Gamma }} \right\rangle }^{{1 \over \Gamma }}}}}} \right)}}} \right]} \right).}}$$

**Step 4.** During this stage, the Method Based on the Removal Effects of Criteria (MEREC) (Keshavarz-Ghorabaee et al., 2021) is employed to determine the weighted vector of attributes. Subsequently, the weighted vector of alternatives is computed following the subsequent steps.

$a.$ Assess the comprehensive performance of alternatives ${{\mathrm{S}}_{\mathrm{i}}}$ by employing the subsequent equation:

(5) $${{\mathrm{S}}_{\mathrm{i}}} = ln\left( {1 + \left( {{1 \over m}\sum\limits_{j = 1}^n {{s_{ij}}} } \right)} \right)$$

where ${s_{ij}}$ denotes the score values of the ${Z_{ij}}$.

$b.$ Determine the performance of alternatives by systematically eliminating individual criteria. Let $\acute{{\mathrm{S}}_{{\mathrm{ij}}}}$ represent the overall performance of i-th alternative concerning the removal of j-th criterion. The subsequent equation is employed to perform the calculations in this step:
(6) $$\acute{{\mathrm{S}}_{{\mathrm{ij}}}} = ln\left( {1 + \left( {{1 \over m}\sum\limits_{k,k \ne j:k,j = 1}^n {{s_{ij}}} } \right)} \right).$$
$c.$ Calculate the sum of absolute deviations. In this stage, determine the removal effect of the j-th criterion using the values derived from $a$ and $b$. Denote the effect of removing the j-th criterion as ${{\mathrm{E}}_{\mathrm{j}}}$. Utilize the subsequent formula to derive the values of ${{\mathrm{E}}_{\mathrm{j}}}$:

(7) $${{\mathrm{E}}_{\mathrm{j}}} = \sum\limits_{i = 1}^m {\left| {\acute{{\mathrm{S}}_{{\mathrm{ij}}}} - {{\mathrm{S}}_{\mathrm{i}}}} \right|}.$$

$d$. Establish the ultimate weights for the criteria. In this stage, compute the objective weight of each criterion by utilizing the removal effects ( ${{\mathrm{E}}_{\mathrm{j}}}$) derived from $c$. Subsequently, represent the weight of the j-th criterion as ${w_j}$. The ensuing equation is employed to calculate ${w_j}$:

(8) $${w_j} = {{{{\mathrm{E}}_{\mathrm{j}}}} \over {\sum\limits_{j = 1}^n {{{\mathrm{E}}_{\mathrm{j}}}} }}.$$

**Step 5.** The overall accumulated value $\acute{E}_{i}$ under various attributes ${\hat c_j}$ is obtained by applying the LIVIFAAWA operator to the decision matrix $\widetilde{T_q}$ created in Step 2.

$$\eqalign{{\widetilde{\acute{E}}_{i}} = &  LIVIFAAWA\left(\acute{E_{i1}},\acute{E_{i2}}, \ldots ,\acute{E_{in}}\right) = \mathop \oplus \limits _{j = 1}^n\left({\bar \lambda _j}\acute{E_{ij}}\right)\cr = &  \left( {\left[ {{{\hat s}_{q\left( {1 - {e^{ - {{\left\langle {\sum\limits_{j = 1}^k {{{\bar \lambda }_j}} {{\left( - \log \left(1 - {{\theta _j^ - } \over q}\right)\right)}\Gamma }} \right\rangle }^{{1 \over \Gamma }}}}}} \right)}},{{\hat s}_{q\left( {1 - {e^{ - {{\left\langle {\sum\limits_{j = 1}^k {{{\bar \lambda }_j}} {{\left( - \log \left(1 - {{\theta _j^ + } \over q}\right)\right)}\Gamma }} \right\rangle }^{{1 \over \Gamma }}}}}} \right)}}} \right]} \right.,\cr  & \left. {\left[ {{{\hat s}_{q\left( {{e^{ - {{\left\langle {\sum\limits_{j = 1}^k {{{\bar \lambda }_j}} {{\left( - \log \left({{\beta _j^ - } \over q}\right)\right)}\Gamma }} \right\rangle }^{{1 \over \Gamma }}}}}} \right)}},{{\hat s}_{q\left( {{e^{ - {{\left\langle {\sum\limits_{j = 1}^k {{{\bar \lambda }_j}} {{\left( - \log \left({{\beta _j^ + } \over q}\right)\right)}\Gamma }} \right\rangle }^{{1 \over \Gamma }}}}}} \right)}}} \right]} \right).}$$

or

$$\eqalign{{\widetilde{\acute{E}}_{i}} = &  LIVIFAAWG\left(\acute{E_{i1}},\acute{E_{i2}}, \ldots ,\acute{E_{in}}\right)\cr = &  \left( {\left[ {{{\hat s}_{q\left( {{e^{ - {{\left\langle {\sum\limits_{j = 1}^n {{\lambda _j}} {{\left( - \log \left({{\left(\theta _{ij}^ - \right)} \over q}\right)\right)}\Gamma }} \right\rangle }^{{1 \over \Gamma }}}}}} \right)}},{{\hat s}_{q\left( {{e^{ - {{\left\langle {\sum\limits_{j = 1}^l {{\lambda _{ij}}} {{\left( - \log \left({{{{\left(\theta _{ij}^ + \right)}^{\left(k\right)}}} \over q}\right)\right)}\Gamma }} \right\rangle }^{{1 \over \Gamma }}}}}} \right)}}} \right]} \right.,\cr  & \left. {\left[ {{{\hat s}_{q\left( {1 - {e^{ - {{\left\langle {\sum\limits_{j = 1}^l {{\lambda _j}} {{\left( - \log \left(1 - {{{{\left(\beta _{ij}^ - \right)}^{\left(k\right)}}} \over q}\right)\right)}\Gamma }} \right\rangle }^{{1 \over \Gamma }}}}}} \right)}},{{\hat s}_{q\left( {1 - {e^{ - {{\left\langle {\sum\limits_{j = 1}^l {{\lambda _j}} {{\left( - \log \left(1 - {{{{\left(\beta _{ij}^ + \right)}^{\left(k\right)}}} \over q}\right)\right)}\Gamma }} \right\rangle }^{{1 \over \Gamma }}}}}} \right)}}} \right]} \right).}$$

**Step 6.** Based on the general LIVIF data of $\acute{E}_{i}$ we calculate the score function ${\widetilde {S}}\left(\widetilde{\acute{E}}_{i}\right)$, in order to find the alternatives $\acute{E}_{i}$, in order find perfect choice $\acute{E}_{i}$. If score functions ${\widetilde {S}}\left({\widetilde{\acute{E}}_\alpha }\right)$ and ${\widetilde{S}}\left({\widetilde{\acute{E}}_\beta }\right)$ are found equally, In light of general LIVIF information for $\acute{E_\alpha }$ and $\acute{E_\beta }$, we continue to determine accuracy degrees ${\widetilde H}\left({\widetilde{\acute{E}}_\alpha }\right)$ and ${\widetilde{H}}\left({\widetilde{\acute{E}}_\beta }\right)$.

**Step 7.** All of the ${\widetilde{S}}\left({\widetilde{\acute{E}}}_{i}\right)$ $\left(i = 1,2, \ldots ,m\right)$ score values are ranked in descending order and the most the desirable choice is chosen.

## Numerical example

In this case study, we consider the issue of health facilities in the hospitals of Peshawar city of kpk Pakistan. Since there are several main hospitals in Peshawar city, including Khyber teaching hospital, LRH Hospital and Peshawar health complex *etc*. These hospitals face a shortage of basic medical equipment and a lack of doctors and paramedical staff during the COVID-19 pandemic. Now the government wants these hospitals to be ranked according to their healthcare facilities, so the hospitals lacking health facilities can be improved. To overcome this issue, the following approach may be followed.

Suppose there are four hospitals, $\acute{E}_{1}$, $\acute{E}_{2}$, $\acute{E}_{3}$ and $\acute{E}_{4}$. The criteria for formatting the alternatives are listed below:

1. ${\hat c_1}$: medical equipment’s;
$2$. ${\hat c_2}$: well trained staff;
$3$. ${\hat c_3}$: cost of treatment of patients;
$4$. ${\hat c_4}$:patient recovery rate.

Suppose there are three decision makers $K = \{ {K_1},{K_2},{K_3}\} ,$ who are tasked to evaluate the four alternatives’ core competencies using these four criteria. The LIVIFN ${Z_{ij}} = \left([\hat s_{{\theta _{ij}}}^ - ,\hat s_{{\theta _{ij}}}^ + ],[\hat s_{{\beta _{ij}}}^ - ,\hat s_{{\beta _{ij}}}^ + ]\right)$ can be used to denote the evaluated value of alternative and the decision-maker ${K_t}\left(t = 1,2,3\right)$ using criteria ${\hat c_j}$ where $j = 1,2,3,4.$ The three experts ${K_1},{K_2}$ and ${K_3}$ are allowed to use the linguistic term set and offer their choices for each alternative $\acute{E}_{i}$ on each attribute ${\hat c_j}$. The preferences of the three experts are shown in Tables 2–4 are in the following order:

**Step 1.** The linguistic interval valued decision matrices presented by the DMs are presented in Tables 2–4.

**Table 2:** LIVIF decision matrix $T_q^{\left(1\right)}$.

	$\acute{E}_{1}$	$\acute{E}_{2}$	$\acute{E}_{3}$	$\acute{E}_{4}$
${\hat c_1}$	$\left([\acute{s}_{2},\acute{s}_{4}],[\acute{s}_{1},\acute{s}_{3}]\right)$	$\left([\acute{s}_{1},\acute{s}_{4}],[\acute{s}_{2},\acute{s}_{3}]\right)$	$\left([\acute{s}_{3},\acute{s}_{5}],[\acute{s}_{1},\acute{s}_{2}]\right)$	$\left([\acute{s}_{2},\acute{s}_{4}],[\acute{s}_{2},\acute{s}_{3}]\right)$
${\hat c_2}$	$\left([\acute{s}_{2},\acute{s}_{3}],[\acute{s}_{3},\acute{s}_{5}]\right)$	$\left([\acute{s}_{2},\acute{s}_{4}],[\acute{s}_{2},\acute{s}_{3}]\right)$	$\left([\acute{s}_{4},\acute{s}_{6}],[\acute{s}_{1},\acute{s}_{2}]\right)$	$\left([\acute{s}_{1},\acute{s}_{3}],[\acute{s}_{2},\acute{s}_{4}]\right)$
${\hat c_3}$	$\left([\acute{s}_{1},\acute{s}_{2}],[\acute{s}_{3},\acute{s}_{4}]\right)$	$\left([\acute{s}_{1},\acute{s}_{3}],[\acute{s}_{4},\acute{s}_{5}]\right)$	$\left([\acute{s}_{3},\acute{s}_{5}],[\acute{s}_{1},\acute{s}_{3}]\right)$	$\left([\acute{s}_{1},\acute{s}_{3}],[\acute{s}_{2},\acute{s}_{4}]\right)$
${\hat c_4}$	$\left([\acute{s}_{1},\acute{s}_{4}],[\acute{s}_{1},\acute{s}_{3}]\right)$	$\left([\acute{s}_{2},\acute{s}_{3}],[\acute{s}_{1},\acute{s}_{4}]\right)$	$\left([\acute{s}_{3},\acute{s}_{4}],[\acute{s}_{2},\acute{s}_{3}]\right)$	$\left([\acute{s}_{3},\acute{s}_{5}],[\acute{s}_{2},\acute{s}_{3}]\right)$

**Table 3:** LIVIF decision matrix $T_q^{\left(2\right)}$.

	$\acute{E}_{1}$	$\acute{E}_{2}$	$\acute{E}_{3}$	$\acute{E}_{4}$
${\hat c_1}$	$\left([\acute{s}_{2},\acute{s}_{4}],[\acute{s}_{1},\acute{s}_{3}]\right)$	$\left([\acute{s}_{1},\acute{s}_{3}],[\acute{s}_{3},\acute{s}_{5}]\right)$	$\left([\acute{s}_{1},\acute{s}_{4}],[\acute{s}_{2},\acute{s}_{3}]\right)$	$\left([\widetilde{s}_{2},\widetilde{s}_{3}],[\widetilde{s}_{3},\widetilde{s}_{5}]\right)$
${\hat c_2}$	$\left([\widetilde{s}_{2},\widetilde{s}_{3}],[\widetilde{s}_{4},\widetilde{s}_{5}]\right)$	$\left([\widetilde{s}_{1},\widetilde{s}_{2}],[\widetilde{s}_{3},\widetilde{s}_{4}]\right)$	$\left([\widetilde{s}_{3},\widetilde{s}_{6}],[\widetilde{s}_{1},\widetilde{s}_{2}]\right)$	$\left([\widetilde{s}_{2},\widetilde{s}_{4}],[\widetilde{s}_{2},\widetilde{s}_{3}]\right)$
${\hat c_3}$	$\left([\widetilde{s}_{3},\widetilde{s}_{6}],[\widetilde{s}_{1},\widetilde{s}_{2}]\right)$	$\left([\widetilde{s}_{1},\widetilde{s}_{4}],[\widetilde{s}_{3},\widetilde{s}_{4}]\right)$	$\left([\widetilde{s}_{2},\widetilde{s}_{5}],[\widetilde{s}_{1},\widetilde{s}_{3}]\right)$	$\left([\widetilde{s}_{1},\widetilde{s}_{3}],[\widetilde{s}_{2},\widetilde{s}_{5}]\right)$
${\hat c_4}$	$\left([\widetilde{s}_{1},\widetilde{s}_{4}],[\widetilde{s}_{3},\widetilde{s}_{4}]\right)$	$\left([\widetilde{s}_{3},\widetilde{s}_{5}],[\widetilde{s}_{1},\widetilde{s}_{3}]\right)$	$\left([\widetilde{s}_{3},\widetilde{s}_{6}],[\widetilde{s}_{1},\widetilde{s}_{2}]\right)$	$\left([\widetilde{s}_{3},\widetilde{s}_{5}],[\widetilde{s}_{2},\widetilde{s}_{3}]\right)$

**Table 4:** LIVIF decision matrix $T_q^{\left(3\right)}$.

	$\acute{E}_{1}$	$\acute{E}_{2}$	$\acute{E}_{3}$	$\acute{E}_{4}$
${\hat c_1}$	$\left([\widetilde{s}_{3},\widetilde{s}_{5}],[\widetilde{s}_{1},\widetilde{s}_{3}]\right)$	$\left([\widetilde{s}_{3},\widetilde{s}_{5}],[\widetilde{s}_{1},\widetilde{s}_{2}]\right)$	$\left([\widetilde{s}_{1},\widetilde{s}_{4}],[\widetilde{s}_{2},\widetilde{s}_{4}]\right)$	$\left([\widetilde{s}_{1},\widetilde{s}_{2}],[\widetilde{s}_{3},\widetilde{s}_{4}]\right)$
${\hat c_2}$	$\left([\widetilde{s}_{1},\widetilde{s}_{3}],[\widetilde{s}_{2},\widetilde{s}_{4}]\right)$	$\left([\widetilde{s}_{3},\widetilde{s}_{5}],[\widetilde{s}_{1},\widetilde{s}_{3}]\right)$	$\left([\widetilde{s}_{2},\widetilde{s}_{5}],[\widetilde{s}_{2},\widetilde{s}_{3}]\right)$	$\left([\widetilde{s}_{2},\widetilde{s}_{3}],[\widetilde{s}_{1},\widetilde{s}_{5}]\right)$
${\hat c_3}$	$\left([\widetilde{s}_{2},\widetilde{s}_{5}],[\widetilde{s}_{1},\widetilde{s}_{2}]\right)$	$\left([\widetilde{s}_{3},\widetilde{s}_{4}],[\widetilde{s}_{1},\widetilde{s}_{3}]\right)$	$\left([\widetilde{s}_{3},\widetilde{s}_{5}],[\widetilde{s}_{2},\widetilde{s}_{3}]\right)$	$\left([\widetilde{s}_{2},\widetilde{s}_{3}],[\widetilde{s}_{4},\widetilde{s}_{5}]\right)$
${\hat c_4}$	$\left([\widetilde{s}_{1},\widetilde{s}_{2}],[\widetilde{s}_{2},\widetilde{s}_{4}]\right)$	$\left([\widetilde{s}_{1},\widetilde{s}_{3}],[\widetilde{s}_{3},\widetilde{s}_{5}]\right)$	$\left([\widetilde{s}_{3},\widetilde{s}_{6}],[\widetilde{s}_{1},\widetilde{s}_{2}]\right)$	$\left([\widetilde{s}_{3},\widetilde{s}_{5}],[\widetilde{s}_{1},\widetilde{s}_{2}]\right)$

**Step 2.** In this problem we consider ${\hat c_1}$ as the cost attribute, and the remaining all other attributes are benefit attributes, so we use Eq. (4) to transform the LIVIF decision matrices $T_q^{\left(1\right)},T_q^{\left(2\right)}$ and $T_q^{\left(3\right)}$ into the corresponding normalised matrices ${\widetilde{T}}_q^{\left(1\right)},{\widetilde{T}}_q^{\left(2\right)}$ and ${\widetilde{T}}_q^{\left(3\right)}$ (Tables 5–7).

**Table 5:** Normalized LIVIF decision matrix $\widetilde{T}_q^{\left(1\right)}$.

	$\acute{E}_{1}$	$\acute{E}_{2}$	$\acute{E}_{3}$	$\acute{E}_{4}$
${\hat c_1}$	$\left([{\hat s_1},{\hat s_3}],[{\hat s_2},{\hat s_4}]\right)$	$\left([{\hat s_2},{\hat s_3}],[\widetilde{s}_{1},\widetilde{s}_{4}]\right)$	$\left([\widetilde{s}_{1},\widetilde{s}_{2}],[\widetilde{s}_{3},\widetilde{s}_{5}]\right)$	$\left([\widetilde{s}_{2},\widetilde{s}_{3}],[\widetilde{s}_{2},\widetilde{s}_{4}]\right)$
${\hat c_2}$	$\left([\widetilde{s}_{2},\widetilde{s}_{3}],[\widetilde{s}_{3},\widetilde{s}_{5}]\right)$	$\left([\widetilde{s}_{2},\widetilde{s}_{4}],[\widetilde{s}_{2},\widetilde{s}_{3}]\right)$	$\left([\widetilde{s}_{4},\widetilde{s}_{6}],[\widetilde{s}_{1},\widetilde{s}_{2}]\right)$	$\left([\widetilde{s}_{1},\widetilde{s}_{3}],[\widetilde{s}_{2},\widetilde{s}_{4}]\right)$
${\hat c_3}$	$\left([\widetilde{s}_{1},\widetilde{s}_{2}],[\widetilde{s}_{3},\widetilde{s}_{4}]\right)$	$\left([\widetilde{s}_{1},\widetilde{s}_{3}],[\widetilde{s}_{4},\widetilde{s}_{5}]\right)$	$\left([\widetilde{s}_{3},\widetilde{s}_{5}],[\widetilde{s}_{1},\widetilde{s}_{3}]\right)$	$\left([\widetilde{s}_{1},\widetilde{s}_{3}],[\widetilde{s}_{2},\widetilde{s}_{4}]\right)$
${\hat c_4}$	$\left([\widetilde{s}_{1},\widetilde{s}_{4}],[\widetilde{s}_{1},\widetilde{s}_{3}]\right)$	$\left([\widetilde{s}_{2},\widetilde{s}_{3}],[\widetilde{s}_{1},\widetilde{s}_{4}]\right)$	$\left([\widetilde{s}_{3},\widetilde{s}_{4}],[\widetilde{s}_{2},\widetilde{s}_{3}]\right)$	$\left([\widetilde{s}_{3},\widetilde{s}_{5}],[\widetilde{s}_{2},\widetilde{s}_{3}]\right)$

**Table 6:** Normalized LIVIF decision matrix $\widetilde{T}_q^{\left(2\right)}$.

	$\acute{E}_{1}$	$\acute{E}_{2}$	$\acute{E}_{3}$	$\acute{E}_{4}$
${\hat c_1}$	$\left([\widetilde{s}_{1},\widetilde{s}_{3}],[\widetilde{s}_{2},\widetilde{s}_{4}]\right)$	$\left([\widetilde{s}_{3},\widetilde{s}_{5}],[\widetilde{s}_{1},\widetilde{s}_{3}]\right)$	$\left([\widetilde{s}_{2},\widetilde{s}_{3}],[\widetilde{s}_{1},\widetilde{s}_{4}]\right)$	$\left([\widetilde{s}_{3},\widetilde{s}_{5}],[\widetilde{s}_{2},\widetilde{s}_{3}]\right)$
${\hat c_2}$	$\left([\widetilde{s}_{2},\widetilde{s}_{3}],[\widetilde{s}_{4},\widetilde{s}_{5}]\right)$	$\left([\widetilde{s}_{1},\widetilde{s}_{2}],[\widetilde{s}_{3},\widetilde{s}_{4}]\right)$	$\left([\widetilde{s}_{3},\widetilde{s}_{6}],[\widetilde{s}_{1},\widetilde{s}_{2}]\right)$	$\left([\widetilde{s}_{2},\widetilde{s}_{4}],[\widetilde{s}_{2},\widetilde{s}_{3}]\right)$
${\hat c_3}$	$\left([\widetilde{s}_{3},\widetilde{s}_{6}],[\widetilde{s}_{1},\widetilde{s}_{2}]\right)$	$\left([\widetilde{s}_{1},\widetilde{s}_{4}],[\widetilde{s}_{3},\widetilde{s}_{4}]\right)$	$\left([\widetilde{s}_{2},\widetilde{s}_{5}],[\widetilde{s}_{1},\widetilde{s}_{3}]\right)$	$\left([\widetilde{s}_{1},\widetilde{s}_{3}],[\widetilde{s}_{2},\widetilde{s}_{5}]\right)$
${\hat c_4}$	$\left([\widetilde{s}_{1},\widetilde{s}_{4}],[\widetilde{s}_{3},\widetilde{s}_{4}]\right)$	$\left([\widetilde{s}_{3},\widetilde{s}_{5}],[\widetilde{s}_{1},\widetilde{s}_{3}]\right)$	$\left([\widetilde{s}_{3},\widetilde{s}_{6}],[\widetilde{s}_{1},\widetilde{s}_{2}]\right)$	$\left([\widetilde{s}_{3},\widetilde{s}_{5}],[\widetilde{s}_{2},\widetilde{s}_{3}]\right)$

**Table 7:** Normalized LIVIF decision matrix $\widetilde{T}_q^{\left(3\right)}$.

	$\acute{E}_{1}$	$\acute{E}_{2}$	$\acute{E}_{3}$	$\acute{E}_{4}$
${\hat c_1}$	$\left([\widetilde{s}_{1},\widetilde{s}_{3}],[\widetilde{s}_{3},\widetilde{s}_{5}]\right)$	$\left([\widetilde{s}_{1},\widetilde{s}_{2}],[\widetilde{s}_{3},\widetilde{s}_{5}]\right)$	$\left([\widetilde{s}_{2},\widetilde{s}_{4}],[\widetilde{s}_{1},\widetilde{s}_{4}]\right)$	$\left([\widetilde{s}_{3},\widetilde{s}_{4}],[\widetilde{s}_{1},\widetilde{s}_{2}]\right)$
${\hat c_2}$	$\left([\widetilde{s}_{1},\widetilde{s}_{3}],[\widetilde{s}_{2},\widetilde{s}_{4}]\right)$	$\left([\widetilde{s}_{3},\widetilde{s}_{5}],[\widetilde{s}_{1},\widetilde{s}_{3}]\right)$	$\left([\widetilde{s}_{2},\widetilde{s}_{5}],[\widetilde{s}_{2},\widetilde{s}_{3}]\right)$	$\left([\widetilde{s}_{2},\widetilde{s}_{3}],[\widetilde{s}_{1},\widetilde{s}_{5}]\right)$
${\hat c_3}$	$\left([\widetilde{s}_{2},\widetilde{s}_{5}],[\widetilde{s}_{1},\widetilde{s}_{2}]\right)$	$\left([\widetilde{s}_{3},\widetilde{s}_{4}],[\widetilde{s}_{1},\widetilde{s}_{3}]\right)$	$\left([\widetilde{s}_{3},\widetilde{s}_{5}],[\widetilde{s}_{2},\widetilde{s}_{3}]\right)$	$\left([\widetilde{s}_{2},\widetilde{s}_{3}],[\widetilde{s}_{4},\widetilde{s}_{5}]\right)$
${\hat c_4}$	$\left([\widetilde{s}_{1},\widetilde{s}_{2}],[\widetilde{s}_{2},\widetilde{s}_{4}]\right)$	$\left([\widetilde{s}_{1},\widetilde{s}_{3}],[\widetilde{s}_{3},\widetilde{s}_{5}]\right)$	$\left([\widetilde{s}_{3},\widetilde{s}_{6}],[\widetilde{s}_{1},\widetilde{s}_{2}]\right)$	$\left([\widetilde{s}_{3},\widetilde{s}_{5}],[\widetilde{s}_{1},\widetilde{s}_{2}]\right)$

Moreover, in this problem we consider the DMs weight vector $\left({\bar \lambda _1},{\bar \lambda _2},{\bar \lambda _3}\right) = \left(0.3419,0.4121,0.2460\right).$

### LIVIFAAWA operator procedure

**Step 3.** We use the LIVIFAAWA operator with $\Gamma = 1$ and obtain the aggregate matrix $\overline {{T_q}}$ of the three normalized LIVIF decision matrices, as shown in the following Table 8.

**Table 8:** The aggregate LIVIF decision matrix $\overline {{T_q}}$.

	$\acute{E}_{1}$	$\acute{E}_{2}$	$\acute{E}_{3}$	$\acute{E}_{4}$
${\hat c_1}$	$\left([{\hat s_{1.0000}},{\hat s_{3.0000}}],[{\hat s_{2.2097}},{\hat s_{4.2257}}]\right)$	$\left([{\hat s_{2.2192}},{\hat s_{3.7633}}],[\widetilde{s}_{1.3103},\widetilde{s}_{3.7533}]\right)$	$\left([\widetilde{s}_{1.6753},\widetilde{s}_{2.9626}],[\widetilde{s}_{1.4558},\widetilde{s}_{4.3171}]\right)$	$\left([\widetilde{s}_{1.6865},\widetilde{s}_{4.1655}],[\widetilde{s}_{1.6865},\widetilde{s}_{2.9958}]\right)$
${\hat c_2}$	$\left([\widetilde{s}_{1.7681},\widetilde{s}_{3.0000}],[\widetilde{s}_{3.0569},\widetilde{s}_{4.7329}]\right)$	$\left([\widetilde{s}_{1.8869},\widetilde{s}_{3.5955}],[\widetilde{s}_{1.9932},\widetilde{s}_{3.3776}]\right)$	$\left([\widetilde{s}_{3.1547},\widetilde{s}_{5.7902}],[\widetilde{s}_{1.1859},\widetilde{s}_{2.2097}]\right)$	$\left([\widetilde{s}_{1.6753},\widetilde{s}_{3.4393}],[\widetilde{s}_{1.6865},\widetilde{s}_{3.7533}]\right)$
${\hat c_3}$	$\left([\widetilde{s}_{2.1331},\widetilde{s}_{4.7828}],[\widetilde{s}_{1.4558},\widetilde{s}_{2.5348}]\right)$	$\left([\widetilde{s}_{1.5561},\widetilde{s}_{3.6828}],[\widetilde{s}_{2.5262},\widetilde{s}_{4.0222}]\right)$	$\left([\widetilde{s}_{2.5384},\widetilde{s}_{5.0000}],[\widetilde{s}_{1.2605},\widetilde{s}_{3.0000}]\right)$	$\left([\widetilde{s}_{1.2605},\widetilde{s}_{3.0000}],[\widetilde{s}_{2.3718},\widetilde{s}_{4.6327}]\right)$
${\hat c_4}$	$\left([\widetilde{s}_{1.0000},\widetilde{s}_{3.5804}],[\widetilde{s}_{1.8649},\widetilde{s}_{3.6253}]\right)$	$\left([\widetilde{s}_{1.9932},\widetilde{s}_{3.7029}],[\widetilde{s}_{1.5561},\widetilde{s}_{3.9143}]\right)$	$\left([\widetilde{s}_{3.0000},\widetilde{s}_{5.2233}],[\widetilde{s}_{1.3594},\widetilde{s}_{2.3625}]\right)$	$\left([\widetilde{s}_{3.0000},\widetilde{s}_{5.0000}],[\widetilde{s}_{1.6865},\widetilde{s}_{2.7152}]\right)$

**Step 4.** Utilizing (Eqs. (5)–(8)) we obtain the weighted vector of each criteria as:

$({\bar \lambda _1},{\bar \lambda _2},{\bar \lambda _3}$, ${\bar \lambda _4}) = \left(0.2397,0.2476,.2489,0.2639\right).$

**Step 5.** Using the LIVIFAAWA operator with $\Gamma = 1,$ we calculated the total decision values ${\widetilde{\acute{E}}_{i}}$ of each alternative $\acute{E}_{i}$ $\left(i = 1,2, \ldots ,4\right)$ as given below:

$\widetilde{\acute{E}}_{1} = \left([{\hat s_{1.5793}},{\hat s_{3.7734}}],[{\hat s_{2.0099}},{\hat s_{3.5504}}]\right),$

${\widetilde{\acute{E}}}_{2} = \left([{\hat s_{1.9334}},{\hat s_{3.6870}}],[{\hat s_{1.7552}},{\hat s_{3.7505}}]\right),$

${\widetilde{\acute{E}}}_{3} = \left([{\hat s_{2.7034}},{\hat s_{5.0512}}],[{\hat s_{1.5644}},{\hat s_{2.7593}}]\right),$

${\widetilde{\acute{E}}}_{4} = \left([{\hat s_{2.1001}},{\hat s_{4.1014}}],[{\hat s_{1.9405}},{\hat s_{3.3543}}]\right)$.

**Step 6.** We computed the score values $\widetilde{S}\left(\widetilde{\acute{E}}_{i}\right)$ $\left(i = 1,2,3,4\right)$ of the total LIFNs $\widetilde{\acute{E}}_{i}$ $\left(i = 1,2,3,4\right)$, as presented below:

$\widetilde{S}\left(\widetilde{\acute{E}}_{1}\right) = 3.8511,$ $\widetilde{S}\left(\widetilde{\acute{E}}_{2}\right) = 4.0706,$ $\widetilde{S}\left(\widetilde{\acute{E}}_{3}\right) = 4.8867,$ $\widetilde{S}\left(\widetilde{\acute{E}}_{4}\right) = 4.1565.$

**Step 7.** Since $\widetilde{S}\left(\widetilde{\acute{E}}_{3}\right)\, > \,\widetilde{S}\left(\widetilde{\acute{E}}_{4}\right)\, > \,\widetilde{S}\left(\widetilde{\acute{E}}_{2}\right)\, > \,\widetilde{S}\left(\widetilde{\acute{E}}_{1}\right)$ thus we have $\acute{E}_{3}\, > \,\acute{E}_{4}\, > \,\acute{E}_{2}\, > \,\acute{E}_{1}.$ Hence, the best alternative is $\acute{E}_{3}.$

### LIVIFAAWG operator procedure

**Step 3.** We use the LIVIFAAWG operator with $\Gamma = 1$ and obtain the aggregate matrix $\overline {{T_q}}$ of the three normalised LIVIF decision matrices, as shown in the following Table 9.

**Table 9:** The aggregate LIVIF decision matrix $\overline {{T_q}}$.

	$\acute{E}_{1}$	$\acute{E}_{2}$	$\acute{E}_{3}$	$\acute{E}_{4}$
${\hat c_1}$	$\left([{\hat s_{1.0000}},{\hat s_{3.0000}}],[{\hat s_{2.2632}},{\hat s_{4.2733}}]\right)$	$\left([{\hat s_{1.9932}},{\hat s_{3.3514}}],[\widetilde{s}_{1.5561},\widetilde{s}_{3.9143}]\right)$	$\left([\widetilde{s}_{1.5780},\widetilde{s}_{2.8032}],[\widetilde{s}_{1.7606},\widetilde{s}_{4.3747}]\right)$	$\left([\widetilde{s}_{2.6116},\widetilde{s}_{3.9745}],[\widetilde{s}_{1.7681},\widetilde{s}_{3.1547}]\right)$
${\hat c_2}$	$\left([\widetilde{s}_{1.6865},\widetilde{s}_{3.0000}],[\widetilde{s}_{3.2301},\widetilde{s}_{4.7799}]\right)$	$\left([\widetilde{s}_{1.6607},\widetilde{s}_{3.1757}],[\widetilde{s}_{2.5685},\widetilde{s}_{3.7743}]\right)$	$\left([\widetilde{s}_{2.9958},\widetilde{s}_{5.7368}],[\widetilde{s}_{1.2605},\widetilde{s}_{2.2632}]\right)$	$\left([\widetilde{s}_{1.5780},\widetilde{s}_{3.3776}],[\widetilde{s}_{1.7681},\widetilde{s}_{3.9143}]\right)$
${\hat c_3}$	$\left([\widetilde{s}_{1.8649},\widetilde{s}_{3.9404}],[\widetilde{s}_{1.7606},\widetilde{s}_{2.7766}]\right)$	$\left([\widetilde{s}_{1.3103},\widetilde{s}_{3.6253}],[\widetilde{s}_{2.9675},\widetilde{s}_{4.1701}]\right)$	$\left([\widetilde{s}_{2.6098},\widetilde{s}_{5.0000}],[\widetilde{s}_{1.1859},\widetilde{s}_{3.0000}]\right)$	$\left([\widetilde{s}_{1.1859},\widetilde{s}_{3.0000}],[\widetilde{s}_{2.5695},\widetilde{s}_{4.6899}]\right)$
${\hat c_4}$	$\left([\widetilde{s}_{1.0000},\widetilde{s}_{3.3729}],[\widetilde{s}_{2.1331},\widetilde{s}_{3.6828}]\right)$	$\left([\widetilde{s}_{2.2192},\widetilde{s}_{3.9492}],[\widetilde{s}_{1.3103},\widetilde{s}_{3.7533}]\right)$	$\left([\widetilde{s}_{3.0000},\widetilde{s}_{5.4652}],[\widetilde{s}_{1.2674},\widetilde{s}_{2.2973}]\right)$	$\left([\widetilde{s}_{3.0000},\widetilde{s}_{5.0000}],[\widetilde{s}_{1.7681},\widetilde{s}_{2.7706}]\right)$

**Step 4.** Utilizing (Eqs. (5)–(8)) we obtain the weighted vector of each criteria as: $({\bar \lambda _1},{\bar \lambda _2},{\bar \lambda _3}$, ${\bar \lambda _4}) = (0.2589,0.2583,$ $0.2641,0.2188).$

**Step 5.** Applying the LIVIFAAWG operator with $\Gamma = 1$ we calculated the total decision values $\widetilde{\acute{E}}_{i}$ for each alternative $\acute{E}_{i}$ $\left(i = 1,2, \ldots ,4\right)$ as listed below:

$\widetilde{\acute{E}}_{1} = \left([{\widetilde{s}_{1.3183}},\widetilde{s}_{3.2834}],[\widetilde{s}_{2.3349},\widetilde{s}_{4.0448}]\right),$ $\widetilde{\acute{E}}_{2} = \left([\widetilde{s}_{1.7201},\widetilde{s}_{3.4842}],[\widetilde{s}_{2.2973},\widetilde{s}_{4.1720}]\right),$ $\widetilde{\acute{E}}_{3} = \left([\widetilde{s}_{2.3768},\widetilde{s}_{4.2392}],[\widetilde{s}_{1.7093},\widetilde{s}_{3.2607}]\right),$ $\widetilde{\acute{E}}_{4} = ([\widetilde{s}_{1.8289},\widetilde{s}_{3.6377}],$ $[\widetilde{s}_{2.0287},\widetilde{s}_{3.8377}])$.

**Step 6.** We computed the score values $\widetilde{S}\left(\widetilde{\acute{E}}_{i}\right)$ $\left(i = 1,2,3,4\right)$ of the total LIVIFNs $\widetilde{\acute{E}}_{i}$ $\left(i = 1,2,3,4\right)$ listed as $\widetilde{S}\left(\widetilde{\acute{E}}_{1}\right) = 3.5555,$ $\widetilde{S}\left(\widetilde{\acute{E}}_{2}\right) = 3.6838,$ $\widetilde{S}\left(\widetilde{\acute{E}}_{3}\right) = 4.4115,$ $\widetilde{S}\left(\widetilde{\acute{E}}_{4}\right) = 3.9000.$

**Step 7.** Because $\widetilde{S}\left(\widetilde{\acute{E}}_{3}\right)\, > \,\widetilde{S}\left(\widetilde{\acute{E}}_{4}\right)\, > \,\widetilde{S}\left(\widetilde{\acute{E}}_{2}\right)\, > \,\widetilde{S}\left(\widetilde{\acute{E}}_{1}\right)$, therefore $\acute{E}_{3}\, > \,\acute{E}_{4}\, > \,\acute{E}_{2}\, > \,\acute{E}_{1}.$ Hence, the best alternative is $\acute{E}_{3}.$

## Investigation on the impact of parameter $\Gamma$ on DM outcomes

We will rank the alternatives using numerous estimations to characterise working MADM outcomes. The ranking alternatives $\acute{E}_{i}$ $\left(i = 1,2, \ldots ,4\right)$ for the values of $\Gamma$ in the range of $1 \le \Gamma \le 50$ by using the LIVIFAAWA operator are presented in Table 10.

**Table 10:** Ranking order of alternatives $\acute{E}_{i}$ in the range of $1 \le \Gamma \le 50$.

$\lambda$	$\widetilde{S}\left(\widetilde{\acute{E}}_{1}\right)$	$\widetilde{S}\left(\widetilde{\acute{E}}_{2}\right)$	$\widetilde{S}\left(\widetilde{\acute{E}}_{3}\right)$	$\widetilde{S}\left(\widetilde{\acute{E}}_{4}\right)$	Order of alternative
$1$	$3.8511$	$4.0706$	$4.8867$	$4.1565$	$\acute{E}_{3}\, > \,\acute{E}_{4}\, > \,\acute{E}_{2}\, > \,\acute{E}_{1}$
$2$	3.9467	4.0273	4.9241	4.2425	$\acute{E}_{3}\, > \,\acute{E}_{4}\, > \,\acute{E}_{2}\, > \,\acute{E}_{1}$
$3$	4.0367	4.0428	4.9809	4.3229	$\acute{E}_{3}\, > \,\acute{E}_{4}\, > \,\acute{E}_{2}\, > \,\acute{E}_{1}$
$4$	4.1175	4.0573	5.0246	4.3937	$\acute{E}_{3}\, > \,\acute{E}_{4}\, > \,\acute{E}_{1}\, > \,\acute{E}_{2}$
$5$	4.1874	4.0708	5.0591	4.4536	$\acute{E}_{3}\, > \,\acute{E}_{4}\, > \,\acute{E}_{1}\, > \,\acute{E}_{2}$
$6$	4.2465	4.0835	5.0868	4.5034	$\acute{E}_{3}\, > \,\acute{E}_{4}\, > \,\acute{E}_{1}\, > \,\acute{E}_{2}$
$7$	4.2964	4.0953	5.1099	4.5445	$\acute{E}_{3}\, > \,\acute{E}_{4}\, > \,\acute{E}_{1}\, > \,\acute{E}_{2}$
$8$	4.3383	4.1062	5.1295	4.5786	$\acute{E}_{3}\, > \,\acute{E}_{4}\, > \,\acute{E}_{1}\, > \,\acute{E}_{2}$
$9$	4.3736	4.1164	5.1463	4.6073	$\acute{E}_{3}\, > \,\acute{E}_{4}\, > \,\acute{E}_{1}\, > \,\acute{E}_{2}$
$10$	4.4036	4.1258	5.1610	4.6315	$\acute{E}_{3}\, > \,\acute{E}_{4}\, > \,\acute{E}_{1}\, > \,\acute{E}_{2}$
$15$	4.5029	4.1642	5.2136	4.7109	$\acute{E}_{3}\, > \,\acute{E}_{4}\, > \,\acute{E}_{1}\, > \,\acute{E}_{2}$
$20$	4.5574	4.1917	5.2461	4.7545	$\acute{E}_{3}\, > \,\acute{E}_{4}\, > \,\acute{E}_{1}\, > \,\acute{E}_{2}$
$50$	4.6611	4.2609	5.3202	4.8395	$\acute{E}_{3}\, > \,\acute{E}_{4}\, > \,\acute{E}_{1}\, > \,\acute{E}_{2}$

From Table 10, it can be observed that the LIVIFAAWA operator gives the same best choice *i.e.*, $\acute{E}_{3}$, for different values of $\lambda$.

## Comparison analysis

An evaluation of the proposed operators and a few current techniques is presented in this section, such as linguistic intuitionistic fuzzy density hybrid weighted averaging (LIFDHWA) $^{p,q}$ operator (Teng & Liu, 2019), LIVIFHWA operator (Peng, Wang & Cheng, 2018), LIVIFHWG operator (Peng, Wang & Cheng, 2018), LIVIFWA operator (Garg & Kumar, 2019), LIVIFWG operator (Garg & Kumar, 2019). The following Table presents the score values for the options depending on the suggested operators and current techniques.

From Table 11 above, we can see that, by using the linguistic intuitionistic fuzzy density hybrid weighted averaging (LIFDHWA) $^{p,q}$ operator (Teng & Liu, 2019), LIVIFHWA operator (Peng, Wang & Cheng, 2018), LIVIFHWG operator (Peng, Wang & Cheng, 2018), LIVIFWA operator (Garg & Kumar, 2019), LIVIFWG operator (Garg & Kumar, 2019), and the proposed LIVIFAAWA operator and LIVIFAAWG operator have the same best alternative, *i.e*., $\acute{E}_{3}$. From this, we may conclude that linguistic intuitionistic fuzzy density hybrid weighted averaging (LIFDHWA) $^{p,q}$ operator, LIVIFHWA operator, LIVIFHWG operator, LIVIFWA operator, and LIVIFWG operators are particular cases of the suggested LIVIFAAWA operator and LIVIFAAWG operator. As a result, our recommended operators are more generic and applicable extensions compared to existing approaches.

**Table 11:** Comparison analysis with a few prevailing techniques.

Techniques	$\widetilde{S}\left(\widetilde{\acute{E}}_{1}\right)$	$\widetilde{S}\left(\widetilde{\acute{E}}_{2}\right)$	$\widetilde{S}\left(\widetilde{\acute{E}}_{3}\right)$	$\widetilde{S}\left(\widetilde{\acute{E}}_{4}\right)$	Ranking of alternatics
linguistic intuitionistic fuzzy density hybrid weighted averaging (LIFDHWA) $^{p,q}$ operator (Teng & Liu, 2019)	$3.8321$	$3.7002$	$3.8587$	$3.6793$	$\acute{E}_{3}\, > \,\acute{E}_{1}\, > \,\acute{E}_{2}\, > \,\acute{E}_{4}$
LIVIFHWA operator (Peng, Wang & Cheng, 2018)	$3.8096$	$4.0057$	$4.8011$	$4.1243$	$\acute{E}_{3}\, > \,\acute{E}_{4}\, > \,\acute{E}_{2}\, > \,\acute{E}_{1}$
LIVIFHWG operator (Peng, Wang & Cheng, 2018)	$4.3872$	$4.1648$	$3.2591$	$3.9378$	$\acute{E}_{3}\, > \,\acute{E}_{4}\, > \,\acute{E}_{2}\, > \,\acute{E}_{1}$
LIVIFWA operator (Garg & Kumar, 2019)	$3.8540$	$4.0128$	$4.7466$	$4.1281$	$\acute{E}_{3}\, > \,\acute{E}_{4}\, > \,\acute{E}_{2}\, > \,\acute{E}_{1}$
LIVIFWG operator (Garg & Kumar, 2019)	$0.6314$	$3.7291$	$4.5642$	$3.9779$	$\acute{E}_{3}\, > \,\acute{E}_{4}\, > \,\acute{E}_{2}\, > \,\acute{E}_{1}$
Proposed LIVIFAAWA operator	$3.9481$	$4.0287$	$4.8577$	$4.2266$	$\acute{E}_{3}\, > \,\acute{E}_{4}\, > \,\acute{E}_{2}\, > \,\acute{E}_{1}$
Proposed LIVIFAAWG operator	$3.5555$	$3.6838$	$4.4115$	$3.9000$	$\acute{E}_{3}\, > \,\acute{E}_{4}\, > \,\acute{E}_{2}\, > \,\acute{E}_{1}$

## Conclusions

In summary, this study has made significant contributions to the field of decision-making by introducing a novel set of operating principles employing Aczel-Alsina t-norm and t-conorm operators in the context of LIVIF scenarios. These principles have facilitated the development of various new aggregation operators, including the LIVIFAAWA, LIVIFAAOWA, and LIVIFAAHA operators, along with their specialized counterparts, the LIVIFAAWG, LIVIFAAOWG, and LIVIFAAHG operators.

By considering these innovative operators, we have established a comprehensive approach to address MADM challenges within the framework of LIVIF. The practicality of this approach is aptly demonstrated through a mathematical example, underscoring its efficacy in maintaining the quality of decision-making processes.

Furthermore, an examination of Table 11 reveals that, through the utilization of the linguistic intuitionistic fuzzy density hybrid weighted averaging (LIFDHWA) $^{p,q}$ operator (Teng & Liu, 2019), LIVIFHWA operator (Peng, Wang & Cheng, 2018), LIVIFHWG operator (Peng, Wang & Cheng, 2018), LIVIFWA operator (Garg & Kumar, 2019), LIVIFWG operator (Garg & Kumar, 2019), and our proposed LIVIFAAWA and LIVIFAAWG operators, the same best alternative, denoted as $\acute{E}_{3}$, is achieved. Consequently, it may be inferred that the linguistic intuitionistic fuzzy density hybrid weighted averaging (LIFDHWA) $^{p,q}$ operator, LIVIFHWA operator, LIVIFHWG operator, LIVIFWA operator, and LIVIFWG operators are specific instances of the suggested LIVIFAAWA and LIVIFAAWG operators. As a result, our recommended operators demonstrate greater generality and applicability compared to existing approaches.

In future research, we plan to integrate insights from recent studies to advance the application of LIVIF aggregation operators in decision-making processes. Specifically, we aim to incorporate methodologies proposed in recent articles such as pricing policy for a dynamic spectrum allocation scheme (Wu, Jin & Yue, 2022), PAL-BERT, an improved question answering model (Zheng et al., 2024), support vector machine parameter optimization algorithm (Li & Sun, 2020), adapting feature selection algorithms (Liu et al., 2023), an inclusiveness-degree based Signed Deffuant–Weisbush model (Jiang et al., 2024), and others to enhance the adaptability, robustness, and performance of our aggregation operators across various real-world scenarios. By integrating insights from diverse domains, we seek to contribute to the broader research agenda of developing methodologies that bridge theoretical advancements with practical applications in decision support systems.

## Additional Information and Declarations

### Data Availability

The following information was supplied regarding data availability: This is a theory article.
